# Hepatic Mitochondrial Dysfunction and Gut Dysbiosis Induced by Polyethylene Microplastics in FVB/n Mice: A Comparative Study of Fluorescent and Non-Fluorescent Particles

**DOI:** 10.3390/toxics14050386

**Published:** 2026-04-30

**Authors:** Mónica G. Silva, Beatriz Medeiros-Fonseca, Adelina Gama, Isabel Gaivão, Sílvia Nunes, Mariana Fernandes, Paula A. Oliveira, Vicente Monedero, Manuel Zúñiga, Maria Manuel Oliveira, Francisco Peixoto

**Affiliations:** 1 Chemistry Research Centre (CQ-VR), University of Trás-os-Montes and Alto Douro (UTAD), 5000-801 Vila Real, Portugal; mspf@utad.pt (M.F.); mmso@utad.pt (M.M.O.); fpeixoto@utad.pt (F.P.); 2 CITAB—Centre for the Research and Technology of Agro-Environmental and Biological Sciences, University of Trás-os-Montes and Alto Douro (UTAD), 5000-801 Vila Real, Portugal; fonsecabeatriz@live.com.pt (B.M.-F.); pamo@utad.pt (P.A.O.); 3 Animal and Veterinary Research Centre (CECAV), University of Trás-os-Montes and Alto Douro (UTAD), 5000-801 Vila Real, Portugal; agama@utad.pt (A.G.); igaivao@utad.pt (I.G.); 4 Associate Laboratory for Animal and Veterinary Sciences (AL4AnimalS), 1300-477 Lisboa, Portugal; 5 FibEnTech—Fiber Materials and Environmental Technologies, University of Beira Interior, 6200-001 Covilhã, Portugal; silvia.nunes@ubi.pt; 6 Inov4Agro—Institute for Innovation, Capacity Building and Sustainability of Agri-Food Production, 5000-801 Vila Real, Portugal; 7 Instituto de Agroquímica y Tecnología de Alimentos, Calle Agustín Escardino 7, 46980 Paterna, Spain; btcmon@iata.csic.es (V.M.); manolo@iata.csic.es (M.Z.); 8 RISE-Health: Health Research Network, Faculty of Medicine, University of Porto, 4099-002 Porto, Portugal

**Keywords:** microplastics, polyethylene, mitochondria, toxicology, animal models

## Abstract

The emerging problem that microplastics pose to our society is reflected in the exponential growth in investigations devoted to uncovering their toxicological potential in humans. However, these studies present several limitations, one of the most significant being the use of microplastics that do not represent their environmental counterparts. In this study, we evaluated the impact of two types of polyethylene microplastics (27–32 µm)—non-fluorescent and fluorescent—on the liver and intestine, targeting mitochondria. FVB/n mice were subjected to a subacute exposure to two concentrations representative of human exposure (0.002% (*w*/*w*) and 0.006% (*w*/*w*)). Both types of microplastics impaired mitochondrial respiration through disruption of NADH-linked pathways, with more pronounced effects at the highest concentration of fluorescent MPs. Electron transport chain complexes, particularly CIII and CIV, were affected, partially explaining the observed alterations in mitochondrial respiratory capacity. An increased SOD and GPx activity supported the link between mitochondrial dysfunction and increased reactive oxygen species overproduction under MPs exposure. Hepatic mitochondrial lipid remodelling was detected following exposure to fluorescent microplastics, while intestinal epithelial cells displayed impaired mitochondrial activity together with compromised cellular integrity, indicative of stress response. In parallel, shifts in gut composition suggest that PE MPs may contribute to intestinal barrier dysfunction. Overall, fluorescent MPs induced more severe mitochondrial and biochemical disturbances in both the liver and the intestine than their non-fluorescent counterparts. Our findings highlight mitochondria as central targets for microplastic-induced toxicity and underscore the need for improved MPs models in toxicological research.

## 1. Introduction

The global economy and the immense demand for industrial production, coupled with the fast-paced lifestyle characterised by overconsumption and convenience, have led to an unprecedented production of plastics. This rise in plastic production has made plastic waste one of the major environmental problems of our time [1]. Current estimates indicate that global plastic waste has reached 225 million tons per year, corresponding to an average of 28 kg per person [2].

Microplastics (MPs) emerged as a direct consequence of plastic pollution, being considered as ubiquitous particles, sized below 5 mm, with no lower limit established [3]. At the Second Session of the United Nations Environment Assembly (UNEA-2), microplastic pollution was listed as a major environmental and ecological concern [4]. Their ubiquitous distribution and their extremely persistent nature are leading to unavoidable human exposure to microplastics [5,6]. Polyethylene (PE), polystyrene (PS), and polypropylene (PP) are the main chemical components of MPs and are commonly used as models in scientific research [5]. PE has a ubiquitous presence in the consumer market, with a broad range of applications, including packaging, construction, medical materials, and consumer goods, making it a dominant material in daily life. Its recyclability has contributed to its widespread use, but inadequate recycling practices and high production volumes lead to significant environmental persistence [7,8].

The gastrointestinal tract is one of the main targets of MPs’ action, serving as the interface between the body and the external environment [9]. Growing evidence demonstrates that the gut microbiota is a toxicological target of environmental pollutants, with microplastics being no exception. Dysbiosis of intestinal flora, characterised by a depletion of commensal bacteria and an increase of the pathogenic ones, is frequently reported following MPs exposure [10,11]. In fact, this condition is frequently associated with pathological changes, such as inflammatory bowel disease. In addition, MPs can accumulate in the gut, compromising the integrity of the intestinal barrier and increasing its permeability, which may facilitate their translocation into systemic circulation [9,11,12]. Indeed, Zhang and collaborators [13] describe the internalisation of PS particles in the intestinal epithelial cell line IEC-6 following exposure for one and three hours.

Once translocated into the bloodstream, MPs can accumulate in the liver through enterohepatic circulation, as the liver receives around 70% of the portal vein blood drained from the mesenteric veins of the intestinal tract. This close relationship makes the liver one of the organs most prone to suffering from the impacts of MPs, being considered a primary target organ for MPs-induced toxicity [14,15]. Oxidative stress, inflammation, and alterations in lipid metabolism are among the more frequent hepatic effects reported following MPs exposure [14,16,17,18,19].

At the cellular level, *in vitro* studies have demonstrated the internalisation of MPs in different cell lines, suggesting that organelle disruption, such as that of mitochondria, may underlie their toxic effects [20]. Furthermore, several studies have suggested that certain NPs can be colocalised with mitochondria [14,17]. Mitochondria are the core of cellular metabolism, housing oxidative phosphorylation for energy production, and regulating key biosynthetic and catabolic pathways essential for cell function. Due to their central role, they are considered classical targets for stressor-induced toxicity evaluation [21]. Typical effects of MPs-induced mitotoxicity include alterations in mitochondrial architecture and dynamics [22,23], disruption of the mitochondrial membrane [14,24], and changes in ATP levels [20,22].

Mechanistic studies further revealed that MPs can affect the electron transport chain (ETC) and mitochondrial respiration. For instance, Cheng and colleagues [25] found that exposure to aged PP MPs in human-derived liver organoids reduced the enzymatic activity of ETC components, namely Complex I and IV. In the same line of investigation, Yang et al. [26] reported decreased mRNA levels of mitochondrial respiratory complex subunits–Complex I MT-DN1, Complex III MT-CYB, and Complex IV MT-CO1–after exposing lung epithelial cells to PS nanoplastics (NPs), suggesting disrupted mitochondrial function. Furthermore, the authors observed a reduced capacity for oxidative phosphorylation. This was characterised by decreases in basal, maximal, and leak mitochondrial-dependent respiration, typical mitochondrial respiratory states commonly used to evaluate coupling and bioenergetic function, corroborating the impairment of mitochondrial function. Consistent results were reported by Lin et al. [20], who observed suppressed mitochondrial respiration after exposure to both fluorescent and non-fluorescent PS NPs in an *in vitro* model. Although oxidative stress has been implicated in MPs-induced mitochondrial dysfunction [17,26], mitochondrial function can also be modulated by molecular factors such as lipid composition of the mitochondrial membrane [27]. Therefore, approaches aiming to evaluate mitochondrial damage and determine its origin should be developed. Addressing this gap is essential to clarify the mechanisms underlying MPs at the cellular level.

Although *in vivo* studies provide valuable insights into the potential toxicological impacts of MPs in humans, they present several limitations. Fluorescent MPs, which may not accurately represent their environmental counterparts, are widely used in toxicological testing and present one of the most significant limitations [18,28,29,30]. The toxic potential of these particles could differ from that of non-fluorescent, environmentally relevant ones, potentially leading to an overestimation of their harmful effects [31]. Furthermore, despite PE being the most abundant polymer in the environment, most toxicological studies have largely focused on PS. The low density and hydrophobicity of PE present technical challenges for *in vitro* and *in vivo* testing, which may explain its limited use. These discrepancies underscore a critical gap in MPs, emphasising the need for studies employing environmentally relevant conditions. Thus, in this research, we aimed to assess the impacts of two different types of PE MPs—non-fluorescent and fluorescent—to unveil if they possess equivalent toxic effects, with a particular focus on mitochondrial function as the central target of MPs-induced toxicity.

## 2. Materials and Methods

### 2.1. Characterisation of Polyethylene Microplastics

Fluorescent Yellow and Non-Fluorescent Blue Polyethylene Microspheres with sizes of 27–32 µm were purchased from Cospheric (Somis, CA, USA). The morphology and size of PE particles were corroborated by scanning electron microscopy (SEM). The SEM images were obtained at 20 kV on a Hitachi S-3400N type II microscope (Hitachi, Chiyoda, Japan) equipped with a Bruker x-flash 5010 at high vacuum. The sample was coated with gold. The MPs’ composition was confirmed by Attenuated Total Reflection Fourier-Transform Infrared (ATR-FTIR) in an IRAffinity 1s (Shimadzu; Corp. Kyoto, Japan) spectrophotometer. The spectra were collected over the 4000–500 cm^−1^ range by averaging 64 scans at a wavenumber resolution of 2 cm^−1^. Solid samples (2 mg) were finely ground, mixed with approximately 175 mg of dried potassium bromide and pressed into pellets. The hydrodynamic size and zeta potential in HEPES buffer (100 mM) were detected in a dynamic light scattering instrument. (Litesizer 500; Anton Paar, Grz, Austria). This characterization was performed to ensure comparability between particle types and to rule out major physicochemical differences other than fluorescence.

### 2.2. Animals and Microplastic Exposure

#### 2.2.1. Animals and Ethics Statement

Thirty FVB/n male mice (26–32 weeks old) were used for this study. Animals were healthy, non-genetically modified, and had no prior experimental procedures. During the experimental time, animals were housed in Eurostandart, Type 1264C polycarbonate cages (Tecniplast, Buguggiate, Italy) and kept in climate-controlled facilities (temperature 23 ± 2 °C, humidity 50 ± 10%, 12/12 h day/night cycle). Animals were fed a standard laboratory diet (Diet Standard 4RF21^®^, Mucedola, Italy). Food and tap water were provided *ad libitum*. Protocols for all animal experiments were approved by the University of Trás-os-Montes and Alto Douro Ethics Committee (ORBEA) and the Portuguese Veterinary Directorate (DGAV) (approval no. 0421/000/000/2023, approved on 28 March 2023), according to the Portuguese (Artigo 44°, Decreto-Lei n°113/2013, August 7) and European (EU Directive 2010/63/EU) Legislation.

#### 2.2.2. Microplastic Exposure

In this study, both non-fluorescent and fluorescent polyethylene microplastics (PE MPs and f-PE MPs, respectively) were used. The size of MPs is within the size range of MPs detected in human stools and heart [32,33].

The PE MPs and f-PE MPs were administered through the diet. A concentration of 0.002% (*w*/*w*) and 0.006% (*w*/*w*), corresponding to Low (L) and High (H), for both types of MPs was incorporated into the standard chow diet, as described in the Supplementary Materials–Methods section (Method S1). These values present administered doses rather than biologically effective internal exposure, due to the limited understanding of MPs’ toxicokinetics. The thirty mice were randomly divided into 5 experimental groups (6 animals per group, *n* = 6): CTL, PE–L (Low), PE–H (High), f-PE–L (Low) and f-PE–H (High). Based on food consumption records, the daily intake of PE MPs for each mouse was calculated to be 16.1 mg/week/kg body weight (BW) and 44.1 mg/week/kg (BW). When extrapolated to a human weighing approximately 60 kg, this equates to a weekly intake of 0.97 g/week/person and 2.65 g/week/person, respectively. These exposure levels were selected to approximate reported human microplastic ingestion ranges (0.1–5 g/week/person) [34].

Experimental units were randomly allocated to control and treatment groups. The randomisation sequence was generated in Microsoft Excel using a random number function, ensuring unbiased assignment across all groups. To minimise potential confounders, the order of experimental groups was mixed, and cage placement within the rack was varied. Sample size was chosen to provide adequate statistical power while minimising animal use. The primary outcome for sample size calculation was hepatic mitochondrial function. All other measured parameters were treated as secondary outcomes to complement the primary analysis. The animals’ weight, water consumption, and food intake were recorded weekly throughout the experiment. The percentage of ponderal weight gain was calculated by subtracting the initial mouse weight from the final weight and dividing by the initial weight. No humane endpoints were required because dietary microplastic exposure was not expected to cause distress.

Faecal samples were collected from the cages in the final week of the experiment, with the cage considered the experimental unit (*n* = 2 cages per group). After 28 days of MPs exposure, all mice were euthanised by exsanguination via intracardiac punction under anaesthesia (40U, Rompum^®^, Bayer Healthcare S.A., Kiel, Germany), after fasting for 12 h. Blood was collected into plain tubes for biochemical analysis. The liver, kidney, testicle, and heart were collected, weighed, and stored at −80 °C for further analysis. Due to sample availability limitations, serum biochemical and genotoxicity analysis were only performed in a subset of samples (*n* = 3 per group). The exact sample size (*n*) for each analysis is indicated in the corresponding figure and table captions. An aliquot of the liver underwent a preservation process for later high-resolution respirometry evaluation. The intestine was harvested and stored in DPBS at 4 °C for subsequent isolation of the intestinal epithelial cell (IECs) fraction. The protocols followed for the preservation process and isolation of IECs are depicted in the Supplementary Materials (Methods S2 and S3).

### 2.3. Histologic Analysis

Hepatic morphological alterations were assessed by hematoxylin and eosin (H&E) staining. Briefly, the tissue was sectioned, fixed in 10% neutral buffered formalin and embedded in paraffin for pathologic sectioning. Sections of 2 µm were stained with H&E for microscopic observation. Liver was evaluated using the modified Knodell Histological Activity Index (HAI) scoring system to assess the inflammatory activity, by evaluating the presence of: A–Piecemeal necrosis (0–4), B–Confluent necrosis (0–6), C–Focal necrosis, apoptosis and focal inflammation (0–4) and D–Portal inflammation (0–4). The necroinflammatory score results from the sum of A + B + C + D, with a score ≤ 3 indicating mild or no necro-inflammation and ≥10 indicating severe necro-inflammation [35,36].

### 2.4. Serum Biochemistry Analysis

Hepatic damage was evaluated based on serum levels of albumin, aspartate aminotransferase (AST/GOT), and alkaline phosphatase. For this, the collected serum samples were analysed using an automated analyser.

### 2.5. Comet Assay

The conventional alkaline version of the comet assay was followed as described by Collins et al. [37], with an extra step of nucleoid digestion by DNA lesion-specific repair enzyme, formamidopyrimidine DNA glycosylase (FPG), capable of converting oxidised purines and pyrimidines into DNA single strand-breaks, allowing the evaluation of the oxidative damage. A system of two gels per slide was adopted. Four slides precoated with 1% normal melting point agarose were used for each animal, with two replicates.

The cell suspension from the liver (*n* = 3) was obtained by mincing a portion of 5 mg of tissue in 2.5% of mincing solution (HBSS, 2 mM Na_2_EDTA, 10% DMSO). Hepatic cell suspensions were mixed with 1% low-melting-point agarose, and 140 µL of the mixture was placed onto the slide (70 µL for each gel). The slides were placed at 4 °C for 5 min for agarose solidification and prior immersed in a lysis solution (2.5 M NaCl, 100 mM EDTA, 10 mM Tris, 1% Triton X-100, pH 10) for 1 h, at 4 °C. Slides were then rinsed with ice-cold enzyme buffer (100 mM KCl, 0.5 mM EDTA, 40 mM HEPES, 0.2 mg/mL BSA, pH 8.0).

In total, two sets of slides were prepared. One set was used to run the assay with FPG, and the other without it. Hence, 50 µL of 1:10 FPG enzyme was applied to each gel, and only buffer in the remaining set, with coverslips, and incubated for 30 min, at 30 °C in a chamber. Prior, the slides were placed in the electrophoresis tank and immersed in an alkaline electrophoresis solution (0.3 M NaOH, 1 mM EDTA, pH ≥ 13) for 30 min. Electrophoresis was carried out at a voltage of 25 V and a current of 300 mA for 20 min. Gels were then neutralised with PBS and washed with distilled water.

Slides were stained with 4,6-diamidino-2-phenylindole (DAPI), and an Olympus BX41 fluorescent microscope (Olympus, Hachioji, Japan) (400× magnification) was used for DNA visualisation. The nucleoids were classified by visual scoring. From each gel, a total of 100 comets were classified into one of the five categories, each category with a value from 0 to 4, according to the head and tail intensity: 0–undamaged and 4–maximum damage. The total score was expressed as genetic damage index (GDI) and calculated according to the formula:

GDI = [(% nucleoids class 0) × 0] + [(% nucleoids class 1) × 1] + [(% nucleoids class 2) × 2] + [(% nucleoids class 3) × 3] + [(% nucleoids class 4) × 4]

GDI values were expressed on a scale from 0 to 400 arbitrary units. Additional DNA breaks, corresponding to net enzyme-sensitive sites (NSS_FPG_), were calculated by subtracting the enzyme-untreated GDI values from the FPG-treated incubation scores (GDI_FPG_).

### 2.6. High-Resolution Respirometry Measurements

#### 2.6.1. Tissue Processing

Liver tissue was thawed from cryopreservation medium and rinsed with respiration medium (220 mM mannitol, 75 mM sucrose, 0.5 mM EGTA, 0.3 mM magnesium chloride (MgCl_2_), 10 mM KH_2_PO_4_, 10 mM HEPES, 0.1% BSA (*w*/*v*), pH 7.4). A portion of 50 mg of tissue was homogenised in 0.2% (*w*/*v*) ice-cold respiratory medium. Protein concentration in the homogenate was determined directly at 280 nm using a Microvolume μDrop Plate (Thermo Fisher Scientific, Waltham, MA, USA).

#### 2.6.2. Substrate Uncoupler Inhibitor Titration (SUIT) Protocols

Mitochondrial respiration was assessed using an oxygraph-2k high-resolution respirometer (OROBOROS^®^ Instruments; Innsbruck, Austria) at 37 °C, by the sequential additions of substrates, inhibitors, and uncouplers. 

One to five milligrams of protein from tissue homogenate were added to both chambers containing respiratory medium. Oxygen concentration and flow rate were continuously monitored in real-time using the Oroboros DatLab 7.0 software. ROUTINE respiration was assessed after stabilisation of the oxygen flux to a steady state, where the oxygen consumption was constant. Additional measurements were performed under defined non-physiological respiratory states to characterise mitochondrial function. The non-phosphorylating mitochondrial respiratory state (LEAK respiration, *L*–classic state 2) supported by electron flow through Complex I (*L_CI_*) was evaluated by mitochondria energisation by 5 mM pyruvate and 1 mM malate. Saturating concentrations of ADP (1–5 mM) were used for coupling electron transfer to ADP phosphorylation, assessing the oxidative phosphorylation capacity (OXPHOS capacity, *P*–classic state 3) supported by Complex I (*P_CI_*). To test inner mitochondrial membrane integrity, cytochrome *c* (10 µM) was added to the respiratory chamber. The lack of a significant rise in respiratory flux after cytochrome *c* addition suggests that the outer mitochondrial membranes are predominantly intact. 

Succinate (5 mM), a Complex II substrate, was then added to obtain maximal phosphorylating capacity supported by both Complex I+II (*P_CI+II_*), followed by the addition of Complex I inhibitor, rotenone (*P_CII_*). LEAK respiration in the presence of adenylates (ADP) and inhibition of ATP synthase was then assessed by the addition of 5 µM oligomycin (*L_Omy CII_*). Mitochondrial respiration was then completely inhibited by adding Complex III inhibitor antimycin A, to assess residual oxygen consumption (ROX). ROX represents non-mitochondrial oxygen consumption and was subtracted from all respiratory states for data normalisation [38]. The low values obtained for ROX demonstrate the mitochondrial origin of oxygen consumption. Finally, 1 mM TMPD (*N*,*N*,*N*′,*N*′–tetramethyl-p-phenylenediamine) and 2 mM ascorbate were added to the chamber for assessment of non-phosphorylating respiration supported by Complex IV (*L_Omy CIV_*). Subsequently, the oxygen consumption linked to the autoxidation of ascorbate and TMPD was evaluated through the addition of Complex IV inhibitor, sodium azide (40 mM). The oxygen concentration in the chambers was maintained above the safe limit of oxygen to avoid respiratory limitation (90 nmol O_2_/mL) [39]. Classical respiratory control ratios (RCR) were not used as primary indicators of coupling efficiency, as freezing is known to partially disrupt the inner mitochondrial membrane integrity [40]. Results were normalised by mitochondrial mass, reflected by citrate synthase activity, and expressed as pmol O_2_/CS activity.

### 2.7. Mitochondrial Enzymes Evaluation

#### 2.7.1. Isolation of Mitochondrial Fractions

Mitochondrial fractions were obtained by differential centrifugation as described by Peixoto et al. [41]. A portion of the liver was weighed and added to ice-cold isolation medium (250 mM sucrose, 0.2 mM EGTA, 0.1 mM EDTA and 0.1% BSA, pH 7.0). IECs were recovered from the storage medium by centrifugation and then resuspended in the isolation medium. Following mechanical homogenization, both homogenates were centrifuged at 800× *g* for 10 min at 4 °C. The obtained supernatant was centrifuged at 10,000× *g* for 10 min at 4 °C for mitochondria sedimentation. For IECs, both the late supernatant and the pellet obtained from the initial centrifugation cycle were reserved for oxidative stress evaluation. Mitochondrial fraction was washed twice and resuspended in washing medium (250 mM sucrose, 5 mM HEPES, pH 7.4) at a protein concentration of 10–20 mg/mL for liver samples and 2–4 mg/mL for IECs, respectively. Total protein concentration was assessed by the Biuret method [42], using a BSA standard curve.

#### 2.7.2. Mitochondrial Enzymatic Activities Assessment

Mitochondrial fractions underwent three cycles of freezing and thawing to disrupt the mitochondria. Kinetic reactions were performed for 5 min, at 30 °C, in a microplate reader (Multiskan™ Sky-High Microplate Spectrophotometer, Thermo Fisher Scientific, Waltham, MA, USA), using 0.1–0.2 mg protein of the mitochondria-rich fraction. All assays were performed as described by Spinazzi et al. [43], with modifications. Results were normalised through citrate synthase (CS) activity.

Mitochondrial content was assessed through evaluation of citrate synthase activity, following the reduction of 5,5-dithio-bis-2-nitrobenzoic acid (DTNB) (ε = 13.6 mM^−1^⋅cm^−1^) in a medium containing Tris buffer (200 mM Tris, 0.2% Triton X-100, pH 8.0), mitochondrial fraction, 0.1 mM DTNB, and 0.37 mM Acetyl-CoA, at 412 nm. The kinetic reaction was started by the addition of 0.5 mM oxaloacetate. Enzymatic activity of CS was present as µM DTNB reduced⋅min^−1^⋅mg^−1^ protein.

Complex I (NADH dehydrogenase) activity was evaluated at 340 nm following nicotinamide adenine nucleotide (NADH) oxidation (ε = 6.2 mM^−1^⋅cm^−1^). The reaction was started by adding 0.015 mM of decylubiquinone (DUB) to a microplate well containing phosphate buffer (50 mM KH_2_PO_4_, pH 7.5), sample, 2 mg/mL BSA, 0.3 mM potassium cyanide (KCN) and 0.2 mM NADH. For the assessment of Complex I-specific activity, a parallel assay was conducted in the presence of 0.0125 mM rotenone, a specific inhibitor of Complex I. Results were expressed as µM NADH oxidised⋅min^−1^⋅mg^−1^ protein.

Enzymatic activity of Complex II (succinate dehydrogenase) was calculated by 2,6-dichlorophenolindophenol (DCPIP) reduction (ε = 19.1 mM^−1^⋅cm^−1^) at 600 nm, in a medium containing phosphate buffer (50 mM KH_2_PO_4_, pH 7.5), mitochondrial fraction, 1 mg/mL BSA, 0.3 mM KCN, 20 mM succinate, and 0.075 mM DCPIP. The addition of 0.05 mM DUB started the kinetic reaction. Complex II activity was expressed as µM DCPIP reduced⋅min^−1^⋅mg^−1^ protein. Only malonate-sensitive activity was considered.

Complex II+III activity was evaluated following the reduction of oxidised cytochrome *c* (ε = 29.5 mM^−1^⋅cm^−1^) at 550 nm. This reaction is promoted by Complex III (Coenzyme Q–cytochrome *c*-oxireductase) with cytochrome *c* oxidised acting as an electron acceptor from succinate oxidation through the action of Complex II. The enzymatic assay was performed at 550 nm by adding 8 mM succinate to a well containing phosphate buffer (50 mM KH_2_PO_4_, pH 7.5), sample, 0.1 mM KCN, and 0.5 mM rotenone. The addition of 16 µM cytochrome *c* oxidised started the kinetic reaction. Results were expressed as µM cytochrome *c* reduced⋅min^−1^⋅mg^−1^ protein. Only antimycin A-sensitive activity was considered.

The oxidation of reduced cytochrome *c* was followed at 550 nm for the determination of Complex IV (Cytochrome *c* oxidase) enzymatic activity in medium with phosphate buffer (100 mM KH_2_PO_4_, pH 7.0), mitochondrial fraction, 0.01 mM antimycin A and 0.012 mM rotenone. The addition of 28 µM reduced cytochrome *c* started the kinetic reaction. To evaluate the specific activity of Complex IV, a parallel assay was performed in the same conditions with 1.25 mM KCN, a selective inhibitor of Complex IV. Results were expressed as µM cytochrome *c* oxidised⋅min^−1^⋅mg^−1^ protein.

### 2.8. Oxidative Stress Evaluation

#### 2.8.1. Sample Processing

Samples were processed according to Silva et al. [44] with short modifications. Briefly, a portion of liver was added to 2% (*w*/*v*) ice-cold homogenization buffer (50 mM KH_2_PO_4_, pH 7.0). The mixture was homogenised and subjected to a cycle of sonication for 2 min (6 pulses of 20 s, 70 A). The cytoplasmic fraction of IECs, reserved during mitochondrial isolation, was subjected to the same cycle of sonication. Three cycles of differential centrifugations were performed at 4 °C (first: 1500× *g*, 10 min; second: 8000× *g*, 10 min; third: 14,000× *g*, 10 min), to obtain cytoplasm and both cellular and mitochondrial fractions. The protein supernatant collected after the final centrifugation, representing the cytoplasmic fraction, was used to assess enzyme activity and reduced (GSH) and oxidised (GSSG) glutathione content. Lipid peroxidation (LPO) levels were evaluated in the resultant pellets from the different centrifugation steps, representing both cellular and mitochondrial lipid fractions. Total protein levels of supernatant and pellets were measured following the Biuret method [42]. Cytoplasm fractions were analysed immediately, and lipid fractions were stored at −20 °C until further analysis.

#### 2.8.2. Antioxidant, Biotransformation, and Metabolic Enzyme Activities

Enzymatic activities were evaluated using a microplate reader (Multiskan™ Sky-High Microplate Spectrophotometer, Thermo Fisher Scientific, Waltham, MA, USA). Kinetic reactions were monitored for 3 min and performed at 30 °C. All assays were performed according to Silva et al. [44], with minor modifications.

Catalase (CAT) activity was determined at 240 nm following the reduction of hydrogen peroxide (H_2_O_2_) (ε = 43.6 mM^−1^⋅cm^−1^) to oxygen and water, in the presence of potassium phosphate buffer (50 mM KH_2_PO_4_, pH 7.0), sample, and 9 mM of H_2_O_2_. Enzymatic activity of CAT was represented as mM H_2_O_2_ reduced·min^−1^·mg^−1^ protein. Total and mitochondrial superoxide dismutase (t-SOD and mt-SOD, respectively) were evaluated using the xanthine-xanthine oxidase system at 560 nm. To a well containing potassium phosphate buffer (50 mM KH_2_PO_4_, 1 mM EDTA, pH 7.4), a volume of cytoplasmic fraction, 0.1 mM hypoxanthine, and 0.05 mM nitroblue tetrazolium chloride (NBT) were added. The addition of 28 mU/mL xanthine oxidase initiated the hydroxylation of hypoxanthine, generating superoxide anion. In a parallel assay, 0.25 mM KCN was added for mt-SOD evaluation. A control assay, without a sample, was conducted to obtain maximum NBT reduction by superoxide. Results were expressed as U⋅min^−1^⋅mg^−1^ protein, where one unit was defined as the amount of SOD needed to inhibit 50% of NBT reduction to formazan. Glutathione peroxidase (GPx) activity was evaluated indirectly, at 340 nm, by following the nicotinamide-adenine dinucleotide phosphate oxidation (NADPH) (ε = 6.2 mM^−1^⋅cm^−1^) by glutathione reductase (GR) action. The reaction mixture contained potassium phosphate buffer (100 mM KH_2_PO_4_, 1 mM EDTA, pH 7.0), sample, 5 mM sodium azide (NaN_3_), 1 mM GSH, 1 mM NADPH, and 1.8 U/mL GR. The addition of 0.882 mM H_2_O_2_ acted as a reaction starter. GPx activity was reported as µM NADPH oxidised⋅min^−1^⋅mg^−1^ protein. GR activity was determined by monitoring NADPH oxidation at 340 nm in a well containing potassium phosphate buffer (100 mM KH_2_PO_4_, 0.5 mM EDTA, pH 7.4), cytoplasmic fraction, 0.5 mM NADPH and 1 mM of oxidised glutathione responsible for the kinetic reaction start. Enzymatic activity was expressed as µM NADPH oxidised⋅min^−1^⋅mg^−1^ protein.

The biotransformation enzyme glutathione-*S*-transferase (GST) was assessed at 340 nm following the conjugation of GSH (1 mM) with 2,4-dinitrochlorobenzene (CDNB, ε = 9.6 mM^−1^⋅cm^−1^) (0.5 mM) in a well with potassium phosphate buffer (100 mM KH_2_PO_4_, pH 7.0) and sample. Results were expressed as mM CDNB conjugated⋅min^−1^⋅mg^−1^ protein. Lactate dehydrogenase (LDH) activity was measured at 340 nm, following NADH (0.18 mM, ε = 6.2 mM^−1^⋅cm^−1^) oxidation in the conversion of pyruvate (2 mM) to lactate. Enzymatic activity was represented as mM NADH oxidised⋅min^−1^⋅mg^−1^ protein.

#### 2.8.3. Lipid Peroxidation and Glutathione Content Assessment

Lipid peroxidation was evaluated by measuring thiobarbituric acid reactive substances (TBARS), following Ottolenghi et al. [45], with modifications from Silva et al. [44]. One mL of thiobarcituric acid (TBA) reagent (37.5% (*w*/*v*) trichloroacetic acid (TCA), 0.38% (*w*/*v*) TBA and 0.015% (*w*/*v*) butylated hydroxytoluene (BHT)) was added to cellular and mitochondrial lipid fractions, and incubated at 100 °C for 15 min. The mixture was cooled on ice and centrifuged at 1600× *g* for 10 min at 4 °C. The supernatant was collected, and the absorbance was measured at 532 nm. Lipid peroxidation was estimated as malondialdehyde (MDA) levels using the molar extinction coefficient of 1.56 × 10^5^ M^−1^·cm^−1^. Results were expressed in µM MDA⋅mg^−1^ protein.

The reduced (GSH) and oxidised glutathione ratio (GSSG) was assessed for redox state evaluation, according to Hissin and Hilf [46], with adaptations by Silva et al. [44], using the fluorochrome *ortho*-phthalaldehyde (OPT). Fluorescence was read at excitation at 339 nm and emission at 426 nm, in an Agilent Varian Cary Eclipse Fluorescence Spectrophotometer (Agilent Technologies, Santa Clara, CA, USA). For GSH levels measurement, cytoplasmatic fractions with potassium phosphate buffer (100 mM KH_2_PO_4_, 5 mM EDTA, pH 8) and OPT (200 µL, 1 mg/mL ethanol) were incubated in the dark, for 15 min at room temperature. GSSG concentrations were assessed by incubating the samples with 0.7 mM *N*-ethylmaleimede (NEM) and 3.26 mM sodium hydroxide (NaOH, pH 12.0) for 30 min, followed by the addition of 9.4 mM NaOH. The mixture was then incubated for 15 min at room temperature, in the dark. Calibration curves were obtained for GSH and GSSG concentration estimation, with results being expressed as their ratio (GSH/GSSG).

### 2.9. Hepatic Lipid Profile Evaluation

#### 2.9.1. Lipid Crude Isolation

Total lipids of mitochondria-rich fractions were extracted according to the modified method of Bligh and Dyer [47]. Lipid crude extraction was performed using methanol/chloroform/water (2:1:0.8, *v*/*v*/*v*). Water was added to 3 mg of mitochondrial protein to a final volume of 0.8 mL, followed by 2 mL of methanol, under agitation for 5 min. One mL of chloroform was added, and the mixture was agitated for another 5 min and then centrifuged at 2000× *g* for 5 min. The pellet was discharged, and 1 mL of chloroform and H_2_O were added to the supernatant following a vigorous vortex. To obtain a two-phase system, samples were centrifuged at 2000× *g* for 5 min. The organic phase (bottom one) was isolated for lipid collection, dried under a nitrogen stream, and stored at −20 °C until further analysis.

#### 2.9.2. Phospholipid Quantification Assay

The phosphorus assay described by Bartlett and Lewis [48] was used to quantify membrane phospholipid (PL) content. To 50 µL of mitochondrial lipid extract, previously dried under a nitrogen stream, a volume of 650 µL of perchloric acid (70%) was added and then incubated for 2 h at 180 °C. A volume of 3300 µL of H_2_O, 500 µL of ammonium molybdate (10% *w*/*v*) and 500 µL ascorbic acid (2.5% *w*/*v*) were added to the mix and incubated at 100 °C for 10 min. A calibration curve of phosphate standard was prepared and underwent the sample treatment. Absorbance was measured at 800 nm in a Multiskan™ Sky-High Microplate Spectrophotometer (Thermo Fisher Scientific, Waltham, MA, USA).

#### 2.9.3. Separation of Phospholipid Classes by Thin-Layer Chromatography (TLC)

Phospholipid (PL) classes from lipid crude were separated by thin layer chromatography (TLC) using silica gel 60 plates with a concentration zone (2.5 × 20 cm) [49]. Plates were pre-washed with chloroform/methanol (1:1, *v*/*v*), air dried, activated with 2.3% (*w*/*v*) boric acid in ethanol, and dried in an oven at 100 °C for 15 min. Lipid extracts were applied in spots across the concentration zone of the plate in a volume corresponding to 0.5–2 µg of phospholipids, dried in nitrogen flow, and developed in a solvent mixture of chloroform/ethanol/water/triethylamine (30:35:7:35, *v*/*v*/*v*/*v*). Phospholipid spots were revealed with a primuline solution (0.5 mg/mL in acetone/water, 80:20, *v*/*v*) and detected with a UV lamp (254 nm). After spot identification by comparison with phospholipid standards, spots were scraped off from the plates, and the different PL classes were further quantified using the phosphorus assay. The relative abundance of each phospholipid class was determined by calculating the ratio of the phosphorus content in each spot to the total phosphorus content in the sample.

#### 2.9.4. Fatty Acid Analysis

Fatty acid methyl esters (FAME) were obtained by base-catalysed transmethylation of 10 µmol of lipid-enriched fractions, previously added to a glass tube and dried under a nitrogen stream [50]. Methylated C19:0 internal standard in hexane (3 µg/mL) and 200 µL of a methanolic solution of potassium hydroxide (2 M) were added to the tubes. After vigorous vortex agitation for 2 min, 2 mL of NaCl solution (10 g/L) was added, followed by centrifugation for 5 min at 900× *g*. A volume of 500 µL of the organic phase was collected, and the FAME sample was completely dried under a nitrogen stream. GC-MS analysis was performed using a Trace GC Ultra with a Polaris Q mass spectrometer (ThermoScientific, Waltham, MA, USA). The mass spectrometer was operated at full scan mode. Separation was carried out in a DB-FFAP column (30 m × 0.32 mm, 0.25 µm film thickness) with a flow rate of 1.4 mL/min of helium as carrier gas. FAME were redissolved in 100 µL of hexane, and 2 µL of the sample were injected with a CombiPAL autosampler (CTC Analytics AG, Zwingen, Switzerland) in splitless mode, with a splitless time of 1 min. The injector and ion source temperatures were set at 220 °C. The oven temperature program was 58 °C for 2 min, followed by a linear increase to 160 °C at 25 °C/min, then raised by 2 °C/min until 210 °C, finally increased to 225 °C at 20 °C/min, and held at that temperature for 20 min. Data acquisition and results treatment were performed with Xcalibur data system 2.0 (ThermoScientific, Waltham, MA, USA). Fatty acid (FA) identification was carried out by comparing their retention time and by matching their mass spectral fragmentation profile with commercial FAME standards (Supelco 37 Component FAME Mix; Sigma-Aldrich, St. Louis, MO, USA). The relative abundance of FA was calculated by integrating the area under each corresponding peak, dividing by the sum of all areas of identified FA, without correction factors. Results were normalised to the area of the internal standard (C19:0). The peroxidability index (PI) and double bond index (DBI) were calculated as described elsewhere [51].

### 2.10. Microbiome Analysis

Faecal pellets collected during the final week of the experiment were used for total DNA extraction using the QIAmp DNA stool Mini Kit (Qiagen, Hilden, Germany), following the manufacturer’s protocol. The isolated DNA was subsequently quantified using a Qubit 2.0 fluorometer (Invitrogen, Thermo Fisher Scientific, Madrid, Spain) with the dsDNA HS Assay Kit (Invitrogen, Thermo Fisher Scientific, Madrid, Spain). Full-length 16S rDNA gene sequencing was performed using PacBio HiFi technology. The complete bacterial 16S rDNA gene (~1.5 kb) was amplified using universal primers targeting the V1-V9 regions (27F: 5′-AGAGTTTGATCMTGGCTCAG-3′ and 1492R: 5′-GGTTACCTTGTTACGACTT-3′) and sequenced using the Kinnex™ 16S rRNA Kit (PacBio, Menlo Park, CA, USA). Quality assessment was performed using fastp (v.1.0.0) [52], and the Circular Consensus Sequences (CCS) routine was used to generate HiFi reads. Between 47,525 to 84,684 sequences were obtained per sample, with a total number of 2,860,378 reads. DNA amplification and sequencing were performed at FISABIO (Foundation for the Promotion of Health and Biomedical Research of the Valencian Community), Valencia, Spain.

All downstream analyses were conducted accordingly to Domene et al. [53], with modifications, using R software (v4.4.3). The high-throughput sequencing data generated were processed with the DADA2 package v1.34.0 [54]. Raw reads were filtered, discarding sequences shorter than 1000 bp and longer than 1600 bp, with a maximum of 2 expected errors (maxEE = 2), and dereplicated, followed by error rate estimation. Denoising was performed using the inferred error model for amplicon sequence variant (ASV) identification. The denoised sequences were tested for putative chimeric sequences, and taxonomy was assigned using SILVA taxonomy database 138.1 [55]. Data were explored using tools implemented into the phyloseq package (v1.50.1) [56]. Graphic outputs were obtained with the package ggplot2 (v 3.5.1) [57]. ASV sequences were aligned using the function from the DECIPHER package (v3.2.0) [58]. Subsequently, a phylogenetic tree was constructed, with the phangorn package (v2.12.1) [59]. using a maximum likelihood method—the general time reversible model with optimisation of both proportion of invariant sites and gamma-distributed rate heterogeneity (GTR+G+I).

Subsequently, alpha and beta diversity were assessed. For alpha diversity, species richness and diversity estimates were calculated using the breakaway (v4.8.4) [60] and DivNet (0.4.0) [61] packages, respectively. For beta diversity assessment, zCompositions (v1.5.0.5) [62] was used to impute values using the square root Bayesian multiplicative method, replacing the zero counts. A phylogenetic isometric log-ratio transformation was conducted using the philr (1.34.0) [63] package. Euclidean distances were computed from the transformed data. Principal Coordinates Analysis (PCoA) and Non-metric Multidimensional Scaling (NMDS) ordinations were obtained using the tools implemented in phyloseq, based on the previously calculated Euclidean distance matrix. To estimate differences in microbiome among different treatments, pairwise PERMANOVA was performed using the pairwiseAdonis package version 0.4.1 [64], which uses the adonis2 function implemented in package vegan (v2.6-8) [65]. The betadisper and permutest functions implemented in the same package were used to assess the homogeneity of variances. Differential abundance analysis of taxa between groups was performed using beta-binomial count regression models from the package corncob (v0.4.1) [61]. These models account for within-sample taxa correlation and variable sequence depth.

The sequence datasets generated and analysed in this study have been deposited at NCBI (SRR36199943 to SRR36199954) and are available under Bioproject PRJNA1369809.

### 2.11. Statistical Analysis

GraphPad Prism 10 (GraphPad Software, San Diego, CA, USA) and IBM SPSS Statistics version 30.0.0.0 (IBM Corp., Armonk, NY, USA) were used for statistical analysis. Outcome assessments, as well as data analysis, were performed using coded samples, with unblinding occurring only after all analyses were completed.

Data normality was tested using the Shapiro–Wilk test, followed by testing the homogeneity of population variance using the Brown–Forsythe test. For data meeting the assumptions of normality and equal variance, group differences were analysed using one-way ANOVA, followed by Sidak’s post-hoc multiple comparisons test to adjust for multiple testing. Predefined pairwise comparisons included: (1) MPs-treated groups versus the control group, (2) comparisons between fluorescent (f-PE) and non-fluorescent (PE) MPs at the same concentration, and (3) comparisons between low (L—0.002%) and high (H—0.006%) concentrations.

When the assumptions for ANOVA were not met, the Kruskal–Wallis test followed by Dunn’s post-hoc multiple comparisons test was used to control multiple testing. For repeated measures (food and water consumption), a mixed-effects model with Geisser–Greenhouse correction was applied, followed by Tukey’s multiple comparisons test. Histological score distributions were analysed using the chi-square test.

Multiple comparisons were controlled using the corresponding post hoc tests (Sidak’s, Tukey’s, or Dunn’s), as appropriate for each analysis. Statistical significance was set at *p* < 0.05. Outliers were identified using Grubbs’ test (α = 0.05) and excluded from the analysis.

## 3. Results

### 3.1. Characterisation of PE MPs and f-PE MPs

Physicochemical characterisation of both PE MPs and f-PE MPs was performed in this study. FTIR spectra for PE MPs without or with fluorescence are depicted in Figure S1G and Figure S1H, respectively. Both samples are composed of PE chemical structure that consists of repeating C_2_H_4_ units with only alkane-type C–C and C–H bonds. There are no significant differences between the two recorded infrared spectra and the polyethylene spectrum reported in the literature. The main bands and the corresponding attribution are listed in Table S1 [66,67]. Based on SEM analysis, both types of microplastics presented spherical morphology (Figure S1A,B,D,E), and diameters of f-PE MPs and PE MPs had the mean value of 29.4 ± 2.2 µm and 28.3 ± 2.1 µm, respectively (Figure S1C,F). The zeta potential of f-PE MPs and PE MPs was −39.14 mV and −42.83 mV, respectively.

### 3.2. High Concentrations of Fluorescent PE MPs Reduce Weight Gain and Increase Organ Weights in Mice

Animals from the different experimental groups showed lower weight gain than the control group. Still, this depletion was only statistically significant in the group treated with the highest concentration of fluorescent PE MPs (CTL vs. f-PE–H, *p* = 0.0114) (Figure S2C). The relative weight of the liver was similar across all experimental groups. However, the relative organ weights of the heart, testicles, and kidneys differed among the experimental groups. The kidneys and testicles of the animals exposed to the highest concentration of f-PE MPs showed increased relative weight compared to the control group (CTL vs. f-PE–H, *p* = 0.0004 for kidney; *p* = 0.0002 for testicles). A similar trend was observed in the kidneys and heart when comparing the fluorescent and non-fluorescent counterparts (PE–H vs. f-PE–H, *p* = 0.0377 for kidney, *p* = 0.0028 for heart), with organs from animals exposed to f-MPs presenting a higher relative weight. All organs showed increased relative weight when comparing the two concentrations of fluorescent particles (f-PE–L vs. f-PE–H, *p* = 0.0118 for kidney, *p* = 0.0313 for testicles, *p* = 0.0100 for heart) (Table S2).

Water and food consumption showed no statistically significant changes, despite some oscillations in these values during the experimental period (Figure S2A,B).

### 3.3. Low Concentration of PE MPs Triggers AST Increase and Mild Necroinflammatory Changes in Liver

The analysis of serum biomarkers of liver damage (Table 1) showed that PE MPs, at the lowest concentration, significantly increased the levels of aspartate aminotransferase (AST) (CTL vs. PE–L, *p* = 0.0023; PE–L vs. PE–H, *p* = 0.0027; PE–L vs. f-PE–L, *p* = 0.0097), possibly indicating the presence of liver damage following exposure to MPs. However, albumin and alkaline phosphatase values remained similar across all experimental groups.

Table S3 shows liver histological evaluation using the Modified Histological Activity Index (HAI). There were differences in necroinflammatory scores among experimental groups. Although piecemeal and confluent necrosis were mostly absent (score 0) across all groups with no statistical differences, differences were observed for focal necrosis, apoptosis, and focal inflammation (Figure S3). PE–H and f-PE–L groups presented a higher frequency of animals falling into the scores 2 and 3, whereas the CTL group showed mostly scores 0 and 1. In addition, for portal inflammation, the majority of animals presented scores 0–2, with the group f-PE–H presenting a significantly higher number of animals falling in score 1, when compared with control group. Regarding the total necroinflammatory score, animals exposed to MPs tended to exhibit higher scores than controls, but the differences were not statistically significant. A trend towards higher frequencies of scores 2–4 was observed in f-PE MPs groups.

### 3.4. Fluorescent PE MPs Increase DNA Damage Compared to Non-Fluorescent MPs at Low Concentrations

**Figure 1:**
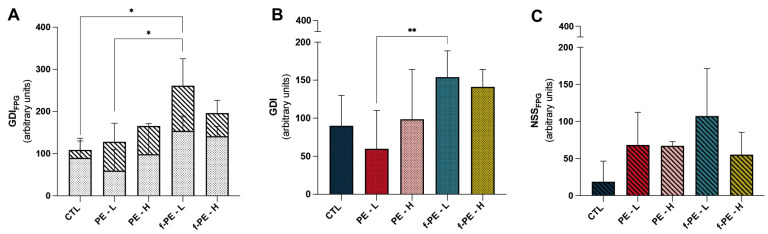
**DNA damage determined by the comet assay in cell suspension from the liver collected from animals exposed to PE-MPs.** Genetic damage index values obtained by the comet assay for (**A**) slides treated with an extra step of digestion formamidopyrimidine DNA glycosylase (FPG) to oxidised purine bases detection (GDI_FPG_), (**B**) untreated slides (GDI), and the resulting (**C**) net FPG-sensitive sites (NSS_FPG_). ***CTL*:** Animals fed standard chow diet without MPs; ***PE–L*:** Animals fed standard chow diet with non-fluorescent PE MPs at 0.002% (*w*/*w*); ***PE–H*:** Animals fed standard chow diet with non-fluorescent PE MPs at 0.006% (*w*/*w*); ***f-PE–L*:** Animals fed standard chow diet with fluorescent PE MPs at 0.002% (*w*/*w*); ***f-PE–H*:** Animals fed standard chow diet with fluorescent PE MPs at 0.006% (*w*/*w*). Values are presented as mean ± SD (*n* = 3, per group) and expressed as arbitrary units. Statistical comparisons between groups are indicated by connecting brackets in the figure. Each bracket represents a predefined pairwise comparison (* *p* < 0.05; ** *p* < 0.01).

Exposure to PE MPs did not induce non-specific DNA damage, reflected by the GDI parameter (Figure 1B). When comparing PE MPs with their fluorescent counterparts, fluorescence increased DNA damage at the lowest concentration of MPs (PE–L vs. f-PE–L, *p* = 0.0080). A similar trend was observed for the GDI_FPG_ parameter (Figure 1A) (PE–L vs. f-PE–L, *p* = 0.0296). Additionally, PE MPs appeared to cause an increase in this parameter, but only with statistical significance for the group f-PE–L (CTL vs. f-PE–L, *p* = 0.0016). No statistical difference was observed regarding oxidative damage despite MP exposure appearing to increase this damage (Figure 1C). Thus, hepatic DNA damage was only present in animals exposed to fluorescence microplastics.

### 3.5. Fluorescent and Non-Fluorescent PE Microplastics Induce Dose- and Type-Dependent Alterations in Mitochondrial Respiration

**Figure 2:**
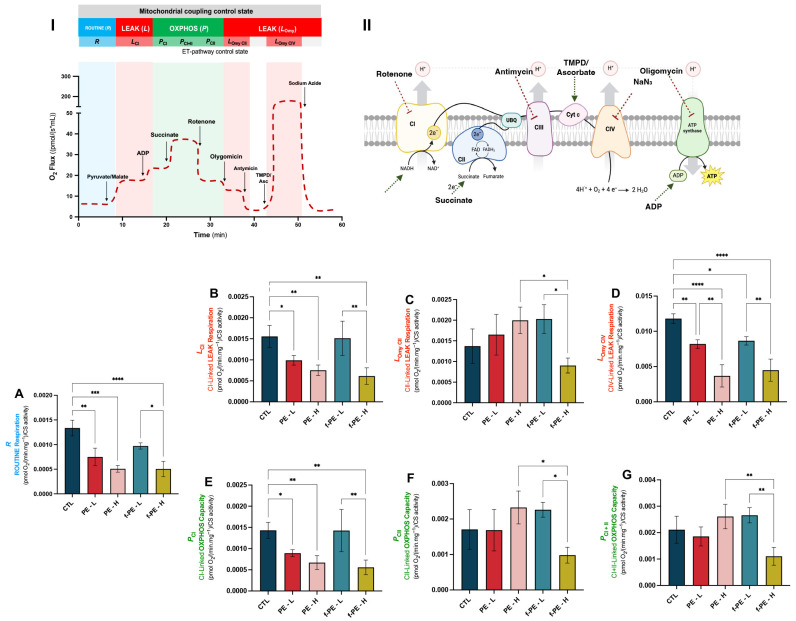
**Assessment of mitochondrial respiration in liver samples from male mice exposed to PE-MPs.** (**I**) Representative respiratory experiment of the substrate uncoupler inhibitor titration (SUIT) protocol used in this study. Bioenergetic parameters were evaluated at baseline ((**A**) ***R*:** ROUTINE respiration, classic State 1) and by sequential addition of 5 mM pyruvate and 1 mM malate ((**B**) ***L*_CI_:** CI-linked LEAK respiration, classic State 2), 1 mM ADP ((**E**) ***P*_CI_:** CI-linked OXPHOS capacity, classic State 3), 5 mM succinate ((**G**) ***P*_CI+II_:** CI+II-linked OXPHOS capacity, classic State 3), 2 µM rotenone ((**F**) ***P*_CII_:** CII-linked OXPHOS capacity, classic State 3), 5 µM oligomycin ((**C**) ***L*_Omy CII_:** CII-Linked LEAK respiration with inhibition of ATP synthase, classic State 4), 5 µM antimycin A (ROX–residual oxygen consumption–non-mitochondrial respiration), 0.5 mM TMPD (+ 2 mM Ascorbate) ((**D**) ***L*_Omy CIV_:** CIV-Linked LEAK respiration with inhibition of ATP synthase, classic State 4) and 20 mM sodium azide (NaN_3_). The mitochondrial coupling state following each titration is indicated above the graph in bold colours (red and green). The ET-pathway control state is shown below the corresponding mitochondrial coupling state in softer colours. (**II**) **Schematic representation of the oxidative phosphorylation system.** Targets of action of the different substrates, uncouplers and inhibitors are depicted. ***CTL*:** Animals fed standard chow diet without MPs; ***PE–L*:** Animals fed standard chow diet with non-fluorescent PE MPs at 0.002% (*w*/*w*); ***PE–H*:** Animals fed standard chow diet with non-fluorescent PE MPs at 0.006% (*w*/*w*); ***f-PE–L*:** Animals fed standard chow diet with fluorescent PE MPs at 0.002% (*w*/*w*); ***f-PE–H*:** Animals fed standard chow diet with fluorescent PE MPs at 0.006% (*w*/*w*). Values are presented as means ± SD (*n* = 6, per group), with two replicates. Statistical comparisons between groups are indicated by connecting brackets in the figure. Each bracket represents a predefined pairwise comparison (* *p* < 0.05; ** *p* < 0.01; *** *p* < 0.001; **** *p* < 0.0001).

Mitochondrial respiration was first allowed to stabilise under baseline conditions, without the addition of substrates, uncouplers, or inhibitors. This initial measurement reflects the ROUTINE respiration, representing the physiological coupling state controlled by endogenous substrate availability and intrinsic energy demands. Results revealed that MPs exposure led to a dose-dependent decrease in ROUTINE respiration, regardless of the type of MPs (Figure 2A). Higher concentrations resulted in a more pronounced reduction in oxygen flux when compared to the control group (CTL vs. PE–L, *p* = 0.0011; CTL vs. PE–H, *p* = 0.0001; CTL vs. f-PE–H, *p* < 0.0001). Comparing both concentrations for the same type of MPs, a decrease is also observed in the highest one, even though this effect is only statistically significant for the fluorescent particles (f-PE–L vs. f-PE–H, *p* = 0.0380).

The non-phosphorylating mitochondrial resting state (LEAK state) supported by CI, CII and CIV was assessed. This mitochondrial coupling control state is characterised by the flux of H^+^ across the inner mitochondrial membrane, in the presence of reducing substrates, due to inherent uncoupling, without using the protonmotive force for ATP synthesis. CI-linked respiration showed the same pattern observed for ROUTINE respiration (CTL vs. PE–L, *p* = 0.0315; CTL vs. PE–H, *p* = 0.0043; CTL vs. f-PE–H, *p* = 0.0011; f-PE–L vs. f-PE–H, *p* = 0.0043) (Figure 2B). A similar trend was observed for LEAK respiration supported by CIV (CTL vs. PE–L, *p* = 0.0071; CTL vs. PE–H, *p* < 0.0001; CTL vs. f-PE–L, *p* = 0.0178; CTL vs. f-PE–H, *p* < 0.0001) (Figure 2D). Additionally, the highest concentration of microplastics resulted in a decrease in oxygen consumption compared to the lowest concentration for both types of microplastics, non-fluorescent (PE–L vs. PE–H, *p* = 0.0052) and fluorescent (f-PE–L vs. f-PE–H, *p* = 0.0043). Results did not follow the same trend when mitochondria were energised with complex II substrate (Figure 2C). The highest concentration of f-PE MPs presented a decrease in LEAK respiration compared to the lowest one (f-PE–L vs. f-PE–H, *p* = 0.0275) and to their non-fluorescent counterparts (PE–H vs. f-PE–H, *p* = 0.0333).

Oxidative phosphorylation (OXPHOS) capacity represents the maximal capacity to generate a protonmotive force for ATP synthesis in the mitochondria. This coupling control state is achieved under conditions of saturated ADP and oxygen concentrations, supported by a substrate or a combination of substrates. Different ET-pathway control states exhibited distinct oxygen flux patterns across the experimental groups. OXPHOS capacity dependent on Complex I energisation presented the same pattern as observed for ROUTINE respiration and CI-linked LEAK respiration (CTL vs. PE–L, *p* = 0.0463; CTL vs. PE–H, *p* = 0.0073; CTL vs. f-PE–H, *p* = 0.0023; f-PE–L vs. f-PE–H, *p* = 0.0062) (Figure 2E).

The highest concentration of fluorescent MPs presented a decreased oxygen consumption rate in respiration supported by CII and CI+II substrates, when compared to the lowest one and their non-fluorescent counterparts (for *P*_CII_: f-PE–L vs. f-PE–H, *p* = 0.0433 for *P*_CII_, *p* = 0.0044 for *P_CI+II_*; PE–H vs. f-PE–H, *p* = 0.0316 for *P*_CII_, *p* = 0.0056 for *P_CI+II_*) (Figure 2F and Figure 2G, respectively).

### 3.6. PE MPs Increase Mitochondrial Content in a Type-Dependent Manner and Differentially Affect the Enzymatic Activities of Mitochondrial Complexes from the Liver

**Figure 3:**
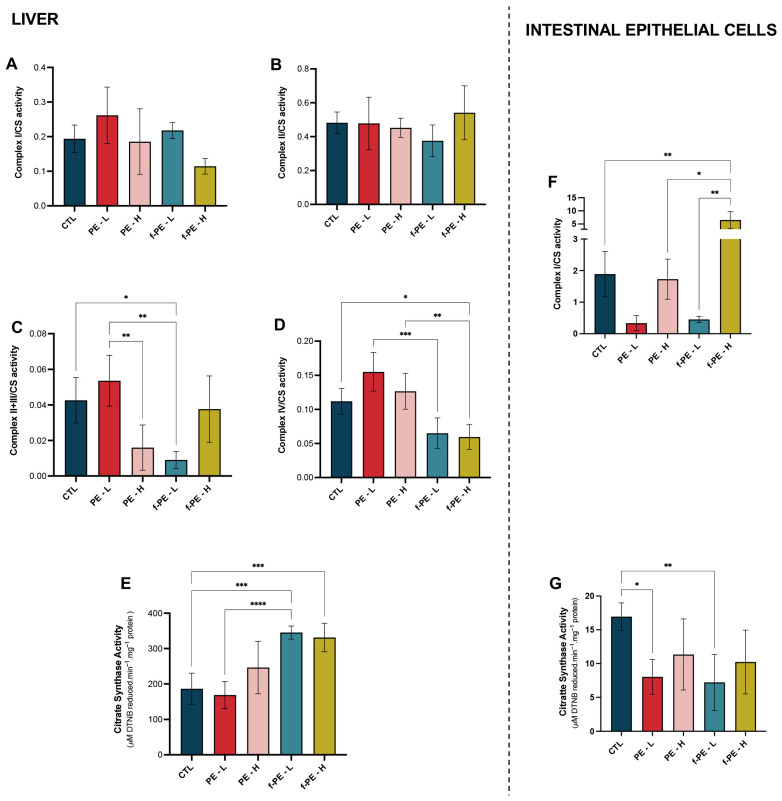
**Effect of two concentrations of fluorescent and non-fluorescent PE-MPs in hepatic and intestinal epithelial cells enzymatic activities of mitochondrial respiratory complexes** (Liver: (**A**) Complex I/CS; (**B**) Complex II/CS; (**C**) Complex II+III/CS; (**D**) Complex IV/CS; Intestinal epithelial cells: (**F**) Complex I/CS) **and citrate synthase** (Liver (**E**) and intestinal epithelial cells (**G**)). Complex activities were normalised with citrate synthase activity. ***CTL*:** Animals fed standard chow diet without MPs; ***PE–L*:** Animals fed standard chow diet with non-fluorescent PE MPs at 0.002% (*w*/*w*); ***PE–H*:** Animals fed standard chow diet with non-fluorescent PE MPs at 0.006% (*w*/*w*); ***f-PE–L*:** Animals fed standard chow diet with fluorescent PE MPs at 0.002% (*w*/*w*); ***f-PE–H*:** Animals fed standard chow diet with fluorescent PE MPs at 0.006% (*w*/*w*). Values are presented as means ± SD (*n* = 6, per group), with two replicates. Statistical comparisons between groups are indicated by connecting brackets in the figure. Each bracket represents a predefined pairwise comparison (* *p* < 0.05; ** *p* < 0.01; *** *p* < 0.001; **** *p* < 0.0001).

The enzymatic activity of mitochondrial complexes and citrate synthase presented different responses to the combinations of type and concentrations of the PE MPs tested. Hepatic mitochondrial isolates from groups treated with fluorescent MPs showed an increase in citrate synthase activity (Figure 3E), a marker of mitochondrial content, compared with the control group (CTL vs. f-PE–L, *p* = 0.0001; CTL vs. f-PE–H, *p* = 0.0002). Complex I and II activity (Figure 3A and Figure 3B, respectively) was not significantly impacted by PE MPs exposure.

In Complex II+III activity (Figure 3C), in the groups exposed to non-fluorescent PE MPs, the highest concentration led to a decreased enzymatic activity when compared to the lowest one (PE–L vs. PE–H, *p* = 0.0052). A similar result was obtained at the lowest concentration of their fluorescent counterparts (PE–L vs. f-PE–L, *p* = 0.0010). Only the group f-PE–L showed a depletion in enzymatic activity compared with the control (*p* = 0.0135).

Fluorescent PE MPs induced a decrease in Complex IV enzymatic activity (Figure 3D) when compared to non-fluorescent PE MPs (PE–L vs. f-PE–L, *p* = 0.0004; PE–H vs. f-PE–H, *p* = 0.0046). PE MPs seem to induce an increase in Complex IV activity, whereas f-PE MPs induced a decrease when compared with control. However, only the decrease observed in mice exposed to f-PE–H presented statistical significance (*p* = 0.0459).

Thus, despite the different responses presented by the complexes evaluated, PE MPs with fluorescence led to a decrease in hepatic complex activities, whereas PE MPs without fluorescence did not seem to affect complex responses when compared to the control.

### 3.7. The Highest Concentrations of PE MPs and f-PE MPs Affect the Hepatic Enzymatic Antioxidant Response, but Do Not Impact Energetic Metabolism

Data on hepatic antioxidant, biotransformation and metabolic enzyme activities are presented in Table 2. Both total and mitochondrial hepatic SOD activities followed a similar pattern across all experimental groups, showing a decrease in response to MPs exposure, slightly more prominent in the fluorescent ones (Total SOD: CTL vs. PE–H, *p* = 0.0360; CTL vs. f-PE–L *p* = 0.0180; CTL vs. f-PE–H, *p* = 0.0010; Mitochondrial SOD: CTL vs. PE–H, *p* = 0.0033; CTL vs. f-PE–L, *p* = 0.0005; CTL vs. f-PE–H, *p* = 0.0002). Similarly, hepatic GR and GPx were also impacted by MPs, showing different responses to the types and concentrations of PE MPs.

Concerning GR activity, the highest concentration of PE MPs increased the enzyme activity compared to the lowest concentration (PE–L vs. PE–H, *p* = 0.0223) (Table 2). However, this pattern was not observed in the groups exposed to f-PE MPs. The highest concentration of fluorescent PE MPs increased GPx activity compared to control and to their non-fluorescent counterparts (CTL vs. f-PE–H, *p* = 0.0438; PE–H vs. f-PE–H, *p* = 0.0031). CAT and GST in the mouse liver remained unchanged following MPs exposure. Lactate dehydrogenase activity remained constant across all experimental groups, indicating that PE MPs probably did not shift the metabolic response.

Thus, exposure to PE MPs, particularly fluorescent ones, altered hepatic antioxidant enzyme activity, suggesting selective oxidative stress responses without major metabolic disruption.

### 3.8. Fluorescent PE MPs Induce Cellular Damage and Disrupt Redox Balance in Hepatic Tissue

The results for cellular damage and redox state assessment in the liver are presented in Table 3. Hepatic mitochondrial and cellular fractions showed different patterns of cellular damage. The mitochondrial fraction did not show any statistically significant changes. Fluorescent PE MPs at 0.006% led to the highest levels of MDA in the cellular fraction compared to the remaining experimental groups (CTL vs. f-PE–H, *p* = 0.0076; PE–H vs. f-PE–H, *p* = 0.0014; f-PE–L vs. f-PE–H, *p* = 0.0108). Thus, the hepatic cellular fraction seems to be more preponed to suffer oxidative damage than the mitochondrial fraction by the fluorescence particles.

Results from the GSH and GSSG ratio in liver samples pointed out that the f-PE–H caused a change in the redox balance, as indicated by the increase observed, but only when compared with the non-fluorescent counterparts (PE–H vs. f-PE–H, *p* = 0.0280).

### 3.9. Hepatic Mitochondrial Lipid Profile Is Selectively Altered by MPs, with Fluorescent MPs Inducing Greater Impact than Non-Fluorescent Counterparts

**Figure 4:**
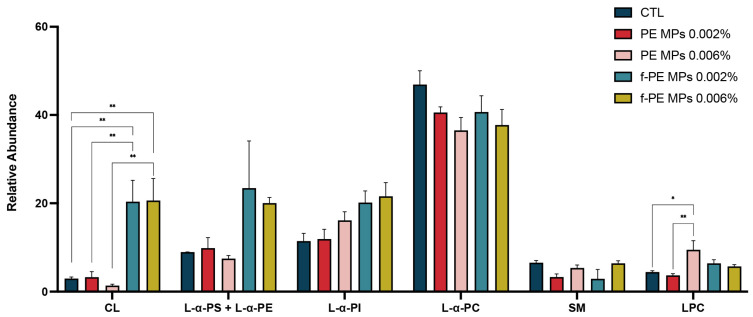
**Impact of PE MPs, with and without fluorescence, on phospholipid composition of mitochondrial-enriched lipidic fractions**. **CL**–Cardiolipin; **L-α-PS**–Phosphatidylserine; **L-α-PE**–Phosphatidylethanolamine; L-**α**-PI–Phosphatidylinositol; SM–Sphingomyelin; **LPC**–Lysophosphatidylcholine. ***CTL*:** Animals fed standard chow diet without MPs; ***PE–L*:** Animals fed standard chow diet with non-fluorescent PE MPs at 0.002% (*w*/*w*); ***PE–H*:** Animals fed standard chow diet with non-fluorescent PE MPs at 0.006% (*w*/*w*); ***f-PE–L*:** Animals fed standard chow diet with fluorescent PE MPs at 0.002% (*w*/*w*); ***f-PE–H*:** Animals fed standard chow diet with fluorescent PE MPs at 0.006% (*w*/*w*). Values are presented as means ± SD (*n* = 6, per group), with two replicates. Statistical comparisons between groups are indicated by connecting brackets in the figure. Each bracket represents a predefined pairwise comparison (* *p* < 0.05; ** *p* < 0.01).

Phospholipid classes—choline phospholipids (phosphatidylcholine (PC), sphingomyelin (SM), and lysophosphatidylcholine (LPC)), phosphatidylethanolamine (PE) combined with phosphatidylserine (PS), phosphatidylinositol (PI) and cardiolipin (CL)—were separated by TLC fractionation and quantified using their relative abundance by the phosphorus assay (Figure 4). PC was the most abundant PL class, followed by PE + PS and PI. As expected in mitochondrial-enriched fractions, SM and LPC were among the least abundant. CL, a key PL in the mitochondrial inner membrane, significantly increased in fluorescent MPs-treated groups compared with control (CTL vs. f-PE–L, *p* = 0.0082; CTL vs. f-PE–H, *p* = 0.0076) and to nonfluorescent counterparts (PE–L vs. f-PE–L, *p* = 0.0052; PE–H vs. f-PE–H, *p* = 0.0048). Although PC, PI, and PS showed no statistically significant differences, PC tended to decrease with MPs exposure, especially at higher concentrations. PE + PS and PI increased with fluorescent MPs. LPC significantly rose in the highest PE MPs exposure group (CTL vs. PE–H, *p* = 0.0145), with a similar but non-significant trend in the fluorescent MPs groups.

**Figure 5:**
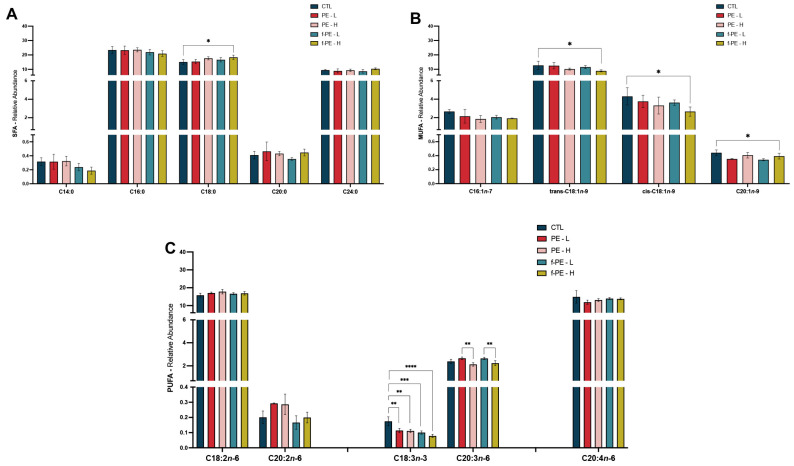
**Impact of microplastics on hepatic mitochondrial membrane lipid profile and relative content:** (**A**) Saturated fatty acids (SFA) relative abundance, (**B**) Monounsaturated fatty acids (MUFA) relative abundance; (**C**) Polyunsaturated fatty acids (PUFA) relative abundance. **C14:0**–Myristic acid; **C16:0**–Palmitic acid; **C16:1**–Palmitoleic acid; **C18:0**–Stearic acid; **trans-C18:1*n*-9**–Elaidic acid; **cis-C18:1*n*-9**–Oleic acid; **C18:2*n*-6**–Linoleic acid; **C18:3*n*-3**–alpha-Linolenic acid; **C20:0**–Arachidic acid; **C20:1*n*-9**–Gondoic acid; **C20:2*n*-6**–Eicosadienoic acid; **C20:3*n*-6**–Eicosatrienoic acid; **C20:4*n*-6**–Arachidonic acid; **C24:0**–Lignoceric acid. ***CTL*:** Animals fed standard chow diet without MPs; ***PE–L*:** Animals fed standard chow diet with non-fluorescent PE MPs at 0.002% (*w*/*w*); ***PE–H*:** Animals fed standard chow diet with non-fluorescent PE MPs at 0.006% (*w*/*w*); ***f-PE–L*:** Animals fed standard chow diet with fluorescent PE MPs at 0.002% (*w*/*w*); ***f-PE–H*:** Animals fed standard chow diet with fluorescent PE MPs at 0.006% (*w*/*w*). Values are presented as means ± SD (*n* = 6, per group), with two replicates. Statistical comparisons between groups are indicated by connecting brackets in the figure. Each bracket represents a predefined pairwise comparison (* *p* < 0.05; ** *p* < 0.01; *** *p* < 0.001; **** *p* < 0.0001).

The hepatic mitochondrial-enriched fraction exhibited a fatty acid profile comprising 14 distinct species: five saturated fatty acid (SFA) species, four monounsaturated fatty acids (MUFA), and five polyunsaturated fatty acids (PUFA) (Figure 5A, Figure 5B and Figure 5C, respectively). Among SFAs, palmitic acid (C16:0) was the most abundant, followed by stearic acid (C18:0) and lignoceric acid (C24:0). Linoleic acid (C18:2*n*-6) was the predominant PUFA and the most abundant *n*-6 species, followed by the arachidonic acid (C20:4*n*-6). The only *n*-3 FA detected was alpha-Linolenic acid (C18:3*n*-3), present at a relative abundance below 1%, similar to that of myristic acid (C14:0), arachidic acid (C20:0), gondoic acid (C20:1*n*-9), and eicosadienoic acid (C20:2*n*-6). Exposure to MPs without fluorescence seems to promote minor changes in the mitochondrial fatty acid profile, with only alpha-Linolenic acid (C18:3*n*-3) presenting a statistically significant decrease when compared with control (CTL vs. PE–L, *p* = 0.0029; CTL vs. PE–H, *p* = 0.0020). Conversely, fluorescent MPs present a more prominent impact in the profile, with the overall impact of fluorescent MPs being the decrease in the content of FA, namely, oleic acid (cis-C18:1*n*-9) (CTL vs. f-PE–H, *p* = 0.0223), elaidic acid (trans-C18:1*n*-9) (CTL vs. f-PE–H, *p* = 0.0377), alpha-Linolenic acid (CTL vs. f-PE–L, *p* = 0.0002; CTL vs. f-PE–H, *p* < 0.0001), and gondoic acid (CTL vs. f-PE–L, *p* = 0.0398). Nonetheless, an increase in stearic acid was also detected following exposure to the highest concentration of fluorescent PE MPs (CTL vs. f-PE–H, *p* = 0.0182).

Quantitative data from Figure 5 were used to calculate total SFA, MUFA, PUFA, *n*-3 and *n*-6 PUFA levels, along with PUFA/SFA and *n*-3/*n*-6 ratios (Table 4). SFA, PUFA and *n*-6 PUFA abundance remained similar across all experimental groups. Conversely, MUFA species presented a decrease in lipid fractions from the liver of animals exposed to MPs. Namely, both types of MPs at the highest concentrations led to a more pronounced decrease in the level of these species compared to the control and the lowest concentration for f-PE MPs (CTL vs. PE–H, *p* = 0.0042; CTL vs. f-PE–H, *p* = 0.0001; f-PE–L vs. f-PE–H, *p* = 0.0109). The levels of *n*-3 PUFA followed a similar trend, presenting a decrease in all MPs-exposed groups (CTL vs. PE–L, *p* = 0.0082; CTL vs. PE–H, *p* = 0.0014; CTL vs. f-PE–L, *p* = 0.0008; CTL vs. f-PE–H, *p* < 0.0001). The trend observed for *n*-3 PUFA resembles that of C18:3*n*-3, which was the only *n*-3 species detected. Exposure to MPs did not lead to significant changes in the double bond and peroxidability index, as so with the PUFA and SFA ratio. A statistically significant decrease of *n*-3/*n*-6 was observed in all MPs-exposed animals (CTL vs. PE–L, *p* = 0.0004; CTL vs. PE–H, *p* < 0.0001; CTL vs. f-PE–L, *p* < 0.0001; CTL vs. f-PE–H, *p* < 0.0001).

### 3.10. MPs Disrupt Mitochondrial Function and Modulate Antioxidant Response in Intestinal Epithelial Cells

IECs mitochondrial fractions exhibited decreased CS activity across all MPs-exposed groups, with a statistically significant reduction at the lowest concentrations (CTL vs. PE–L, *p* = 0.0167; CTL vs. f-PE–L, *p* = 0.0081) (Figure 3G). Complex I was the only mitochondrial complex assessed in IECs’ isolated mitochondria due to sample volume limitations (Figure 3F). Results show that activity was decreased in animals exposed to the lowest concentrations of MPs when compared to the control, despite the absence of statistical significance (CTL vs. PE–L, *p* = 0.8006; CTL vs. PE–H, *p* > 0.9999). The highest concentration of fluorescent MPs presented an increase in CI enzymatic activity compared to their non-fluorescent counterparts and control group (CTL vs. f-PE–H, *p* = 0.0086; PE–H vs. f-PE–H, *p* = 0.0175). These results suggest that both the concentration and type of MPs influence the enzymatic activity of Complex I, potentially leading to malfunction of the ETC.

One of the primary outcomes of ETC dysfunction is an increase in mitochondrial ROS (reactive oxygen species) production, which can contribute to oxidative stress and cellular damage. The antioxidant system of IECs was then assessed. In contrast to the decrease observed in hepatic total SOD activity, enterocyte SOD activity was significantly increased in MPs-exposed groups (CTL vs. PE–L *p* = 0.0002; CTL vs. f-PE–H, *p* < 0.0003) (Table 2). The highest concentration of MPs had an opposite impact on SOD activity: fluorescent particles at the highest concentration led to an increase in SOD activity when compared to the lowest one (f-PE–L vs. f-PE–H, *p* < 0.0001), whereas for the non-fluorescent particles, the highest concentration resulted in a depletion of enzymatic activity (PE–L vs. PE–H, *p* < 0.0001).

CAT and GST activity, similarly to their hepatic counterparts, did not change in animals exposed to MPs compared to the non-exposed ones. Nonetheless, while catalase activity did not differ significantly from the control group, microbeads with and without fluorescence had different effects. Non-fluorescent MPs reduced CAT activity at the highest concentration (PE–L vs. PE–H, *p* = 0.0064), whereas both concentrations of f-PE MPs had a similar impact (Table 2). IECs from animals exposed to MPs presented a loss in GR enzymatic activity (CTL vs. PE–H, *p* < 0.0001; CTL vs. f-PE–L, *p* = 0.0010; CTL vs. f-PE–H, *p* < 0.0001), with a trend towards a greater decrease at higher concentrations (Table 2). GPx activity in MPs-administered groups remained similar to control group. The highest concentration of PE MPs increased the enzymatic activity compared to the lowest (PE–L vs. PE–H, *p* = 0.0065). The presence of fluorescence at the lower concentration tested showed a similar outcome (PE–L vs. f-PE–L, *p* = 0.0026) (Table 2). Lipid peroxidation levels in the IECs’ cellular fractions remained unchanged across all experimental groups (Table 3). An increase in GSH and GSSG ratio was consistently observed in enterocyte samples for all groups exposed to both types and concentrations of MPs (CTL vs. PE–H, *p* = 0.0315; CTL vs. f-PE–L, *p* < 0.0001; CTL vs. f-PE–H, *p* = 0.0218). Overall, MPs exposure led to a modulation of the antioxidant system, indicating that these particles possibly trigger a redox-responsive adaptation, possibly to mitigate an increase in ROS production.

### 3.11. Fluorescent and Non-Fluorescent PE MPs Impact Differently Gut Microbiome Composition

**Figure 6:**
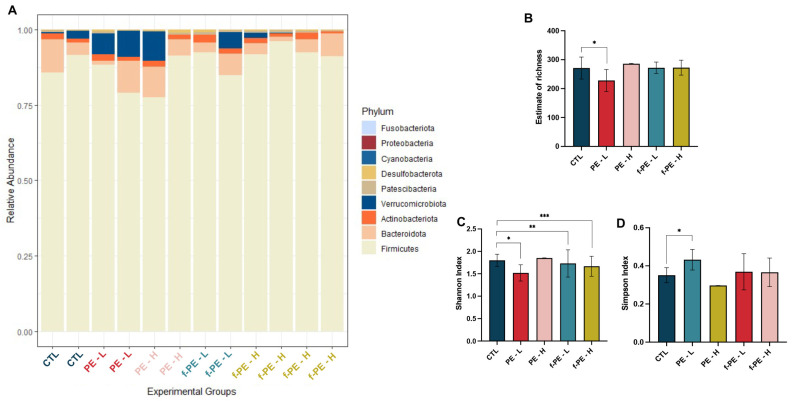
**Microbiome composition analysis of mice exposed to different types and concentrations of PE MPs.** (**A**) Relative abundance of phyla in mice faecal samples; each bar represents a cage faecal sample; (**B**) Estimation of taxonomic richness of faecal samples; (**C**) Estimation of Shannon indexes; (**D**) Estimation of Simpson indexes. Values are means ± SD (*n* = 2) (* *p* < 0.05; ** *p* < 0.01; *** *p* < 0.001).

The impact of polyethylene microplastics with and without fluorescence on intestinal microbiota was evaluated by 16S rDNA sequencing of faecal samples collected from each cage in the last week of the experiment (Week 4). Composition of mice’s microbiome was first assessed at the phylum level, revealing that, as typically described for laboratory mice, *Firmicutes* was the most prevalent taxa, followed by *Bacteroidota, Actinomycetota* and *Verrucomicrobiota* (Figure 6A). The relative abundance of *Verrucomicrobiota* was highly variable both among groups and among samples within each group, with faeces from animals exposed to PE–L presenting the highest levels.

**Figure 7:**
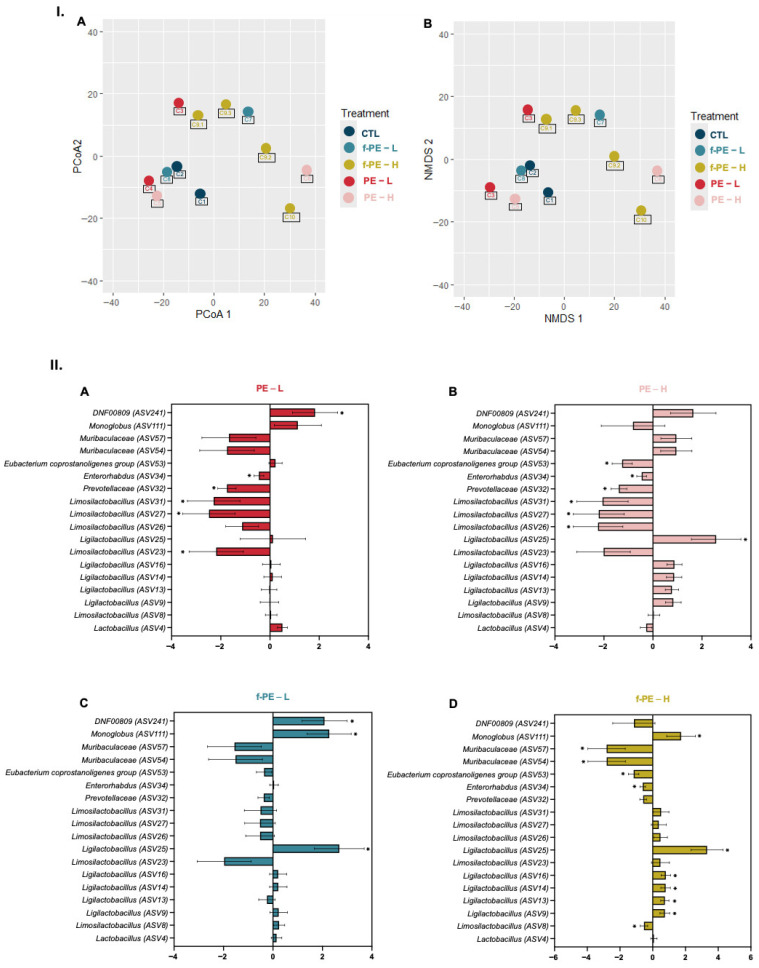
(**I**) **Ordination plots of faecal microbiota composition based on Euclidean distance from isometric log ratio transformed ASV counts** (**A**) Principal coordinates analysis (PCoA) plot. (**B**) Nonmetric multidimensional scaling (NMDS) plot. NMDS ordination of faecal microbiota samples based on the Euclidean distance from isometric log ratio-transformed ASV counts. In the plots, each dot represents the microbiota profile from faecal samples collected from a single cage. Colours represent the different experimental groups: ***CTL*:** Animals fed standard chow diet without MPs; ***PE–L*:** Animals fed standard chow diet with non-fluorescent PE MPs at 0.002% (*w*/*w*); ***PE–H*:** Animals fed standard chow diet with non-fluorescent PE MPs at 0.006% (*w*/*w*); ***f-PE–L*:** Animals fed standard chow diet with fluorescent PE MPs at 0.002% (*w*/*w*); ***f-PE–H*:** Animals fed standard chow diet with fluorescent PE MPs at 0.006% (*w*/*w*). (**II**) **Differential abundance analysis in the faecal microbiota of** (**A**) **PE–L**, (**B**) **PE–H**, (**C**) **f-PE–L and** (**D**) **f-PE–H groups compared to control**. Analysis was conducted by performing beta-binomial regression using the corncob R package. ASVs with FDR-adjusted *p*-values < 0.05 are marked (*). Positive estimates indicate increased abundance, while negative ones indicate decreased abundance in the treatment group.

Alpha diversity analysis at ASV level showed different trends in the groups exposed to MPs. Microbiome samples from mice subjected to the lowest concentration of non-fluorescent PE MPs (PE–L) showed a significant reduction in species richness (Figure 6B). Estimation of Shannon index in the same group showed a similar result (Figure 6C). Groups exposed to fluorescent MPs also displayed a statistically significant decline in this index. Conversely, the Simpson index was increased in PE–L group (Figure 6D). Beta diversity estimations showed that exposure to both types and concentrations of microplastics did not induce significant alterations in the composition of mice microbiota from the different experimental groups. The different ordinations performed, PCoA and NMDS, did not show a clear clustering of samples based on exposure to MPs (Figure 7I(A,B)). Pairwise PERMANOVA corroborated this result, as no significant differences were detected in MPs-exposed groups when compared with the control one. Nonetheless, the analysis of homogeneity of variance, an assumption of PERMANOVA, showed statistical significance, which could be attributed to the low number of samples and the harvesting method.

Following the differences observed in alpha diversity between groups, a differential abundance analysis was conducted to determine which taxa were influenced by MPs exposure. The analysis revealed that a total of eighteen ASVs, representing nine different genera, were significantly influenced by the presence of MPs (Figure 7II(A–D)). Overall, the highest concentration of MPs tested impacted the abundance of a higher number of ASVs, with the effect being most pronounced in the group exposed to fluorescent particles.

PE MPs without fluorescence had a similar effect in differential abundance, at both concentrations, when compared to the control (Figure 7II(A,B)). The genus *Limosilactobacillus* was the most affected, showing a decrease in abundance in both of these groups, with three ASVs significantly changed. Similarly, a depletion of ASV from *Prevotellaceae* and *Enterorhabdus* was observed. In the PE–H group, a decrease in the *[Eubacterium] coprostanoligenes* group was also noted.

Conversely, in the fluorescent MPs exposed groups, different concentrations presented distinct effects on ASV abundance (Figure 7II(C,D)). The lowest concentration of f-PE MPs had a relatively minor impact, with an increase of only three ASVs from the genera *Monoglobus, Ligilactobacilus,* and *DNF00809*. *Ligilactobacillus* abundance was significantly increased in the microbiota of mice exposed to f-PE–H, similar to the alteration observed in the corresponding non-fluorescent concentration. In contrast, a depletion in five ASVs belonging to four different genera—*Limosilactobacillus*, *Enterorhabdus*, *Muribaculaceae*, and *[Eubacterium] coprostanoligenes* group—was reported, similar to that observed in the PE–H group.

## 4. Discussion

The ubiquitous presence of microplastics and their potential health risks have raised significant concerns about their latent adverse effects. Murine models have demonstrated that these particles can cross the intestinal barrier, enter the bloodstream, and accumulate in key metabolic organs [68,69]. Despite growing research on the potential hazards of MPs, current tested models are far from ideal. Using non-fitted models may overestimate the actual risks posed by MPs, leading to inaccurate risk assessments.

In the present study, we selected FVB/n male mice and exposed them to two types of MPs, fluorescent and non-fluorescent, at relevant exposure concentrations for 28 days, representing a subacute exposure [70]. Results showed that, in the tested experimental conditions, PE MPs were associated with alterations in mitochondrial activity, oxidative stress-related parameters, and lipid metabolism, suggesting a potential impact on liver function. In epithelial cells, these MPs may affect mitochondrial health and cellular integrity, potentially contributing to cellular stress. Additionally, changes in gut microbiota suggest that PE MPs may be associated with alterations relevant to intestinal barrier function and systemic metabolism. Overall, fluorescent particles seem to exert a stronger negative impact on both liver and epithelial cell health compared to non-fluorescent counterparts, highlighting their toxic potential and reinforcing their unsuitability for toxicological testing.

Body weight is a typical indicator of overall health. A depletion in body weight or slower weight gain following exposure to MPs has been reported in the literature [11,71]. In our study, animals exposed to the highest concentration of f-PE MPs showed reduced weight gain, despite unchanged food and water consumption, suggesting altered metabolic processes or physiological stress. To evaluate whether this systemic stress extended to organs, we assessed organ coefficients. Most of the organs harvested showed a significant increase in organ coefficient following MPs exposure, except for the liver. These results suggest that MPs can induce systemic effects detectable at both the body and organ levels, but different organs can be affected differently.

MPs may accumulate in the gut, potentially disrupting the intestinal barrier and, in some cases, translocating into systemic circulation, reaching target metabolic organs, such as the liver. Nonetheless, particles at the upper end of the size range, such as those used in this study, are likely to be poorly absorbed. Therefore, the observed effects may not be solely due to direct translocation, and indirect mechanisms, such as alterations in gut barrier integrity, microbiota modulation, or gut–liver axis interactions, may also contribute. At the hepatic level, MPs can both directly and indirectly impact cellular metabolism, leading to energy metabolism disorders, lipid metabolism impairment, and mitochondrial dysfunction [10,17,22]. Liver’s relative organ weight remained unchanged across all experimental groups, despite low to mid hepatic inflammation and necrosis, revealed in histologic analysis, in animals exposed to MPs. Furthermore, serum biochemistry revealed that PE MPs, but only at the lowest concentrations, could significantly increase AST levels, a biomarker of hepatic function, corroborating the presence of tissue damage.

Exposure to MPs has been shown to impair mitochondrial function [14,17,22]. However, most of the studies investigating this effect have employed *in vitro* approaches, with limited to no investigations using *in vivo* models to evaluate MPs-induced mitotoxicity. Mitochondrial respirometry evaluation is considered the hallmark method for assessing mitochondrial dysfunction as it provides a functional assessment of bioenergetics by quantifying oxygen consumption under defined respiratory states [38,72]. To the best of our knowledge, no study has performed hepatic mitochondrial respirometry evaluation following MPs exposure using an *in vivo* model. Findings from *in vitro* studies have been inconsistent, reporting both increases and decreases at different coupling control states. Ma et al. [73] found suppressed basal and maximal respiration in mitochondria isolated from human brain microvascular endothelial cells (hCMEC/D3) and human neuroblastoma cells (SH-SY5Y) following exposure to modified and unmodified PS-NPs. In contrast, Peng et al. [74] observed an increase in all respiratory states in adenocarcinomic human alveolar basal epithelial cells (A549) and adenocarcinomic human epithelial colon cell line (Caco-2) following long-term exposure to fluorescent NPs. These variations suggest that the impact of MPs on mitochondrial bioenergetics is likely influenced by the characteristics of the particles as well as the experimental model used.

In our study, ROUTINE respiration presented a concentration-dependent decrease following exposure to both types of MPs, with CI and CIV-linked LEAK respiration also reduced. Only fluorescent MPs at the highest concentration significantly impaired CII-linked LEAK respiration, suggesting stronger mitotoxic potential. These declines may be associated with dysfunction in mitochondrial complexes or with changes/injuries at the internal mitochondrial membrane (IMM) level, leading to reduced proton pumping, compromising the proton-motive force required for ATP synthesis [75]. Consequently, a loss of mitochondrial membrane potential (MMP) is expected, as reported in different studies [14,20,22]. A decrease in OXPHOS capacity, and consequently an impaired ATP production, has been reported in some studies investigating MPs-induced toxicity [19,20]. Similarly, CI and CII-dependent OXPHOS capacity was decreased. When both substrates were provided simultaneously, the respiratory pattern resembled that of CII alone. This suggests that, at non-physiological conditions, the FADH_2_-dependent respiratory pathway can compensate for the impairment at CI and CIV. However, the reduced routine respiration following MPs exposure highlights that under physiological conditions, mitochondria seem to be dependent on the impaired NADH-linked pathways, and the preservation of CII-dependent respiration does not fully restore the mitochondrial function. Taken together, these findings suggest that MPs may affect mitochondrial respiration *in vivo*, with potential implications for cellular homeostasis, with fluorescent particles exerting stronger effects.

Mitochondrial bioenergetics relies on an intact ETC and sufficient mitochondrial mass [76,77]. To explore whether dysfunction in mitochondrial energy production was associated with impairments at these levels, we assessed the enzymatic activity of individual mitochondrial respiratory complexes and citrate synthase, a key enzyme in the Krebs cycle widely used as a marker of mitochondrial content [78]. Results revealed that f-PE MPs led to an increase in hepatic mitochondrial mass, probably as a compensatory mechanism for downstream dysfunction. Furthermore, exposure to MPs impaired the enzymatic activity of Complexes II+III and IV, with more pronounced effects at higher concentrations and with fluorescent MPs. This decrease could partially impair electron transport efficiency, reducing OXPHOS effectiveness, compromising ATP production, and increasing electron leakage, which in turn may lead to ROS overproduction, consistent with our previous findings [79,80].

Since Complex II remained unaffected, the observed reduction in Complex II+III activity likely reflects alterations in Complex III, one of the major sources of ROS production within the ETC, through the Q-cycle [81,82]. Similar alterations were detected in Complex IV activity, mirroring the pattern observed in CIV-dependent respiration and reinforcing that MPs impaired CIV function, with effects on mitochondrial respiratory capacity. The impact of MPs on mitochondrial complexes has been described previously by Shen al. [19], reporting a decrease in the expression of subunits ND1 and UQCRC2, belonging to CI and CIII, respectively, corroborating the MPs-induced toxicity. Interestingly, CI enzymatic activity remained unaffected, despite the respiratory measurements suggesting dysfunction at this level. Nonetheless, changes in mitochondrial complex activity do not always correlate with mitochondrial respiration [83]. This inconsistency may be justified by the dynamic organisation of the ETC components into supercomplexes, as well as the presence of compensatory mechanisms that enable complexes and supercomplexes to compensate for each other [81,84]. Thus, fluorescent MPs impair specific ETC complexes, particularly CIII and CIV, which compromises OXPHOS capacity and enhances susceptibility to ROS generation.

Oxidative stress is one of the primary mechanisms induced by microplastics, with increased ROS production frequently reported following microplastic exposure [12,18,85]. When ROS generation overcomes the scavenging capacity of the cells’ antioxidant system, it leads to a state of oxidative stress, potentially triggering further mitochondrial and cellular damage [86]. Reduced SOD activity and increased MDA levels are typically associated with MPs-induced oxidative stress, regardless of the type and concentration of MPs tested and the target organ [12,16,87]. These findings are in agreement with our results, where fluorescent MPs at the highest concentration exacerbated oxidative stress. Although no significant differences were detected in mitochondrial MDA levels, probably due to high data variability, the trend towards elevated MDA and a depletion in mitochondrial SOD activity may reflect an overproduction of mitochondrial ROS, probably as a consequence of the impairment observed in the OXPHOS system. This is consistent with the hypothesis that mitochondrial dysfunction may contribute to ROS overproduction under MPs exposure.

Antioxidant enzyme responses were selective. Even though alterations in CAT activity are frequently reported following MPs exposure [12,88], no significant changes were observed in our study. Nonetheless, similar findings were described by Zhang et al. [24] in a study devoted to evaluating the reproductive toxicity of PE-MPs in female mice and their offspring. In contrast, GPx activity increased at the highest concentration of fluorescent MPs. GPx is also capable of neutralising hydrogen peroxide, one of the main ROS species produced within the cell, which may justify the absence of catalase’s response. Moreover, GPx plays a central role in reducing lipidic hydroperoxides, the main products of lipid peroxidation. Thus, the increase in GPx activity could also be interpreted as a response to the increase in cellular MDA levels [18].

Interestingly, the cellular redox state was not impacted by MPs. In fact, at the highest concentration of f-PE MPs, a shift towards a more reductive state was observed when compared to their non-fluorescent counterparts. This aligns with the findings by Cheng and collaborators [25], who attributed this reductive shift to an accumulation of reducing equivalents such as NADH, NADPH, and GSH over their oxidised counterparts. Furthermore, exposure to MPs did not impact intracellular LDH or GST activity, although it induced moderate oxidative stress and mitochondrial impairment. DNA damage was only evident in animals exposed to fluorescent MPs, suggesting that the particles may influence the genotoxic potential, independently of oxidative stress.

Taken together, the results for antioxidant enzymes and cellular damage markers support the hypothesis that MPs-induced mitochondrial dysfunction directly contributes to ROS overproduction, triggering compensatory antioxidant responses, with the liver maintaining its basal metabolic capacity and detoxification potential.

The lipid composition of mitochondrial membranes plays a critical role in regulating mitochondrial function. Phospholipids, the predominant components of mitochondrial membrane bilayers, are vital for maintaining membrane architecture, sustaining mitochondrial dynamics, and ensuring optimal functional capacity [89]. Alterations in PL compositions can directly affect membrane integrity, affecting the stability and activity of IMM-associated proteins, such as those comprising ETC and OXPHOS systems [89]. In mitochondrial membranes, PC represents 40–45% of total lipid content, followed by PE at 25–30%. PI and CL roughly account for 25–30%, while PS represents only 3–5% [90]. Overall, the PL composition of hepatic mitochondrial fractions in our study aligns with literature reports. Minor discrepancies were observed, namely the detection of SM and LPC, which are typically present in mitochondria at levels lower than 1%. Such differences may arise from cross-contamination during mitochondrial isolation via differential centrifugation [91].

Cardiolipin levels were the most impacted by MPs’ exposure. Unlike other phospholipids, CL is exclusively located in mitochondria, mainly within the IMM, and is essential for the structural organisation of ETC supercomplexes and for supporting efficient electron transport. Thus, CL deficiency and/or improper remodelling can impair mitochondrial function. Indeed, a few reports have indicated a decrease in CL levels following MPs exposure for 42 days [92]. Interestingly, in our study, we detected an increase in CL abundance as a consequence of MPs ingestion. This may represent a compensatory response to an external stress, suggesting lipid remodelling to counteract MPs-induced mitotoxicity. Although the mechanisms underlying this response are not clear and evidence is limited, studies have reported an increased or redistributed cardiolipin content in toxicological testing [93,94]. Consistent with stress-induced mitochondrial lipid remodelling, we also observed a significant decrease in PC:PE ratio (results not shown), in the fluorescent MPs-exposed groups, which could be linked to altered bilayer organisation and reduced membrane fluidity [95]. A similar reduction was reported by Chen et al. [16] in mice subjected to long-term large PS MPs exposure, where it was associated with hepatic lipotoxicity. Indeed, toxicological studies have reported that MPs disrupt hepatic lipid metabolism at different levels, including changes in PC and PE content [16,92].

Fatty acids, whether esterified or free, serve multiple functions in cells, acting as structural building blocks of cellular and mitochondrial membranes, energy sources, and signalling molecules [96]. The diversity of saturated, monounsaturated, and polyunsaturated fatty acids allows membranes to fine-tune their fluidity, permeability, and the function of receptors and channels [97]. SFA and PUFA remained constant across all experimental groups, despite a major decrease in alpha-linoleic acid content observed in the groups exposed to MPs. MUFAs were markedly depleted in high concentrations of MPs, particularly in the fluorescent-exposed groups, suggesting a disruption of specific desaturation pathways, potentially favouring the accumulation of saturated species. Indeed, the decreased C18:1*n*-9/C18:0 ratio at the highest concentrations of f-PE MPs (results not shown) suggests a dysfunction of stearoyl coA desaturase (SDC1), the enzyme responsible for stearic acid desaturation [98]. The chain length, degree of unsaturation and double bond position of FA integrated in phospholipids influence the biophysical properties of membranes. MUFAs and PUFAs tend to form more fluid membranes than SFAs [96]. Thus, impaired desaturation capacity, reflected in MUFA depletion, could lead to more rigid mitochondrial membranes. Combined with increased CL content and a lower PC:PE ratio, these changes suggest lipid remodelling under f-PE MPs exposure, as a mechanism to preserve mitochondrial integrity and bioenergetics, given the connection between membrane dynamics, mitochondrial respiration and oxidative stress. Thus, our results suggest that hepatic mitochondria undergo lipid remodelling to cope with f-MPs-induced stress. Nonetheless, despite these compensatory mechanisms, mitochondrial function remains compromised as aforementioned.

Gut epithelial cells, the main functional cells in the intestinal tract, play a critical role in preserving the integrity of the intestinal barrier against external factors. Disturbances at this level can lead to increased intestinal permeability, allowing the translocation of damaging substances, bacteria and macromolecules into the bloodstream, interfering with the host’s health [99,100]. MNPs can affect the intestinal structure and function by increasing intestinal permeability [101], triggering inflammatory responses [28], and inducing oxidative stress [12]. Although MPs did not induce a state of oxidative stress in IECs, the increase in SOD activity in our results suggests elevated ROS production. The antioxidant system of IECs remained functional, yet alterations in redox balance were noted, particularly in groups exposed to higher concentrations of PE MPs and both concentrations of f-PE MPs. These groups showed an increased GSH/GSSG ratio, despite a decrease in GR activity, suggesting redox imbalance. Interestingly, mitochondrial dysfunction, characterised by a reduced mitochondrial content and an increase in Complex I activity at the highest concentrations of MPs, may underline this redox disruption, pointing mitochondria as a potential source of ROS and a key target of MPs-induced toxicity in intestinal epithelial cells. Indeed, a few studies have addressed MPs impact on mitochondria in gut epithelial cells, revealing increased mitophagy and disrupted mitochondrial architecture [102,103]. Overall, f-PE MPs appear to induce more pronounced changes in cellular redox homeostasis and mitochondrial-related parameters compared to pristine PE MPs. Nevertheless, these findings should be interpreted with caution, as they are based on a limited number of evaluated parameters and do not allow firm conclusions regarding intestinal dysfunction or underlying mechanisms.

The presence of MPs in the gastrointestinal tract can significantly impact gut microbial communities, potentially disrupting their composition and functional balance, a condition known as dysbiosis [104]. As frequently reported following exposure to MPs, alpha diversity was impacted [11,105]. A decrease in the Shannon index was observed, reflecting a loss of richness and evenness in the taxa from MPs-exposed groups, a hallmark of dysbiosis. Although beta diversity did not reveal changes in the microbial composition between groups, likely due to limited sample size, differential abundance analysis revealed changes in nine genera. Both concentrations of non-fluorescent MPs reduced ASVs from *the Limosilactobacillus* genus. *Limosilactobacillus* genus, previously belonging to *Lactobacillus*, is crucial for the gut barrier maintenance, antimicrobial activity, and immune modulation [106]. Studies reveal that gut microbiome subjected to toxicological insults, such as the presence of MPs, suffer from a loss of commensal bacteria, such as those belonging to the *Lactobacillaceae* family, often linked to impaired barrier function, inflammation, and oxidative stress [28,102,105]. A decrease in short-chain fatty acids (SCFAs) producing bacteria, such as *Prevotellaceae* and *Enterorhabdus*, was observed, consistent with the findings by Chen et al. [10], suggesting that PE MPs disrupted the balance of the intestinal barrier. Given the known regulatory roles of SCFAs in epithelial protection and mitochondrial function, it is possible that the microbiome shifts observed in this study are related to the mitochondrial alterations detected in IECs. However, these findings should be interpreted with caution, due to the variability and limited depth of our data. These limitations are further compounded by the low number of biological replicates (cage-based sampling, *n* = 2 per group), which reduces statistical power and may limit the robustness of microbiome-related conclusions. Fluorescent PE MPs had a different and more pronounced impact on the gut microbiome than their non-fluorescent counterparts. The highest concentration exerted a stronger effect on ASV abundance, with more taxa affected. Interestingly, one of the main changes at this level was the increased abundance in taxa belonging to the *Ligilactobacillus* genus, functionally similar to *Lactobacillus*. Similar results have been reported across the literature. Qiao et al. [11] showed an increase in *Lactobacillus* in the gut microbiota from C57BL/6J mice exposed to modified and non-modified PS MPs for 28 days, supporting the findings of Li et al. [107] following PE MPs exposure during a similar exposure time. In a similar approach, Fu et al. [108] detected a higher abundance of specimens belonging to the *Lactobacillaceae* family following 10 days of exposure to MPs. These consistent findings suggest that f-PE MPs may promote the growth of *Lactobacillus*-related taxa. However, the functional implications remain to be clarified. Thus, the findings suggest that MPs exposure, particularly at higher concentrations of fluorescent MPs, may be associated with alterations in gut microbiota composition. The functional consequences of these changes, including potential effects on intestinal barrier integrity and host metabolism, remain to be determined.

Overall, our findings demonstrate that fluorescent PE particles, particularly at the highest concentrations, were associated with more pronounced hepatic and intestinal alterations than their non-fluorescent counterparts, with results highlighting mitochondria as a sensitive and relevant target for evaluating MPs-induced toxicity. Nonetheless, this study has some limitations that should be acknowledged. In addition to the inherent limitations associated with toxicological testing of microplastics and the relatively small sample size in some upstream analyses, this study was conducted using only male animals from a single mouse strain. This may limit the generalisability of the findings, as sex- and strain-specific differences in response to MPs exposure cannot be excluded. Therefore, the results should be interpreted with caution, and further studies including both sexes, multiple strains, and expanded endpoints are warranted.

## 5. Conclusions

In summary, our results demonstrate that mitochondria can act as sensitive and relevant targets for evaluating MPs-induced toxicity. Specifically, PE MPs compromise hepatic respiratory capacity, disrupt specific ETC complexes, and trigger compensatory lipid remodelling and antioxidant responses, while also affecting epithelial cell integrity and gut microbiota composition. Fluorescent MPs, particularly at the highest concentrations, had a stronger impact than their non-fluorescent counterparts. Importantly, the concentrations tested were selected to reflect realistic human exposure, unlike previous studies that rely on unrealistically high doses. However, the inherent variability in MPs, due to differences in polymer type, particle size and surface modifications, and experimental setups, makes cross-study comparisons a challenging task and limits definitive conclusions about human health risks. These findings underscore the need for improved MPs models that better reflect environmental particles, alongside standardised guidelines for MPs toxicological assessment, to enable more accurate and reliable risk evaluations.

## Figures and Tables

**Table 1:** **Serum biochemistry analysis of mice exposed to PE-MPs for 28 days. *CTL*:** Animals fed standard chow diet without MPs; ***PE–L*:** Animals fed standard chow diet with non-fluorescent PE MPs at 0.002% (*w*/*w*); ***PE–H*:** Animals fed standard chow diet with non-fluorescent PE MPs at 0.006% (*w*/*w*); ***f-PE–L*:** Animals fed standard chow diet with fluorescent PE MPs at 0.002% (*w*/*w*); ***f-PE–H*:** Animals fed standard chow diet with fluorescent PE MPs at 0.006% (*w*/*w*). In the table, values with different letters indicate statistically significant differences. Values are presented as means ± SD (*n* = 3, per group).

Experimental Group	AST/GOT (UI/L)	Alkaline Phosphatase (UI/L)	Albumin (g/dL)
***CTL***	69.50 ± 12.02 ^a^	61.67 ± 11.24 ^a^	2.87 ± 0.12 ^a^
***PE–L***	444.00 ± 104.65 ^b^	59.33 ± 6.66 ^a^	2.93 ± 0.15 ^a^
***PE–H***	111.00 ± 33.29 ^a^	60.00 ± 7.94 ^a^	3.00 ± 0.10 ^a^
***f-PE–L***	149.50 ± 6.36 ^a^	50.00 ± 8.89 ^a^	2.97 ± 0.06 ^a^
***f-PE–H***	153.67 ± 66.61 ^a^	43.33 ± 11.59 ^a^	2.87 ± 0.25 ^a^

**Table 2:** **Impact of PE MPs in hepatic and intestinal enzymatic antioxidant system** (SOD–superoxide dismutase; CAT–catalase; GR–glutathione reductase; GPx–glutathione peroxidase), (GST–glutathione-*S*-transferase) **and metabolic enzymes** (LDH–lactate dehydrogenase). ***CTL*:** Animals fed standard chow diet without MPs; ***PE–L*:** Animals fed standard chow diet with non-fluorescent PE MPs at 0.002% (*w*/*w*); ***PE–H*:** Animals fed standard chow diet with non-fluorescent PE MPs at 0.006% (*w*/*w*); ***f-PE–L*:** Animals fed standard chow diet with fluorescent PE MPs at 0.002% (*w*/*w*); ***f-PE–H*:** Animals fed standard chow diet with fluorescent PE MPs at 0.006% (*w*/*w*). In the table, values with different letters indicate statistically significant differences. Values are presented as means ± SD (*n* = 6, per group), with two replicates.

	*CTL*	*PE–L*	*PE–H*	*f-PE–L*	*f-PE–H*
***Liver***					
**Total SOD** (U·min^−1^·mg^−1^ protein)	133.60 ± 11.08 ^a^	133.30 ± 13.39 ^a^	111.00 ± 12.42 ^b^	108.90 ± 9.44 ^b^	100.20 ± 9.10 ^b^
**Mitochondrial SOD** (U·min^−1^·mg^−1^ protein)	112.40 ± 6.66 ^a^	113.30 ± 5.29 ^a^	85.70 ± 3.13 ^b^	83.97 ± 7.86 ^b^	80.11 ± 10.85 ^b^
**CAT** (mM H_2_O_2_ reduced·min^−1^·mg^−1^ protein)	67.41 ± 9.87 ^a^	80.21 ± 2.79 ^a^	68.24 ± 10.54 ^a^	65.16 ± 9.13 ^a^	71.75 ± 15.03 ^a^
**GR** (µM NADPH oxidated·min^−1^·mg^−1^ protein)	35.09 ± 11.86 ^a,b^	18.34 ± 4.97 ^a^	46.49 ± 12.00 ^b^	39.74 ± 5.99 ^a,b^	49.55 ± 13.98 ^a,b^
**GPx** (µM NADPH oxidated·mn^−1^·mg^−1^ protein)	128.25 ± 19.83 ^a^	140.75 ± 27.23 ^a,b^	111.63 ± 21.40 ^a^	137.37 ± 7.02 ^a,b^	172.97 ± 27.89 ^b^
**GST** (mM CDN·min^−1^·mg^−1^ protein)	82.04 ± 15.35 ^a^	57.32 ± 38.42 ^a^	48.37 ± 31.81 ^a^	57.11 ± 93.08 ^a^	72.27 ± 50.39 ^a^
**LDH** (mM NADH oxidated·min^−1^·mg^−1^ protein)	0.66 ± 0.09 ^a^	0.63 ± 0.10 ^a^	0.70 ± 0.07 ^a^	0.71 ± 0.06 ^a^	0.74 ± 0.077 ^a^
***Intestinal Epithelial Cells***					
**Total SOD** (U·min^−1^·mg^−1^ protein)	3.30 ± 0.735 ^a,c^	6.23 ± 0.33 ^b^	3.01 ± 0.78 ^a^	4.42 ± 0.66 ^c^	5.88 ± 0.44 ^b^
**CAT** (mM H_2_O_2_ reduced·min^−1^·mg^−1^ protein)	1.83 ± 0.21 ^a,b^	2.29 ± 0.36 ^a^	1.48 ± 0.34 ^b^	1.52 ± 0.20 ^b^	1.56 ± 0.42 ^a,b^
**GR** (µM NADPH oxidated·min^−1^·mg^−1^ protein)	14.36 ± 3.14 ^a^	9.92 ± 2.94 ^a^	5.09 ± 3.00 ^b^	6.41 ± 1.21 ^b^	2.67 ± 0.35 ^b^
**GPx** (µM NADPH oxidated·mn^−1^·mg^−1^ protein)	11.92 ± 2.85 ^a,b^	9.31 ± 2.06 ^a^	16.62 ± 4.62 ^b^	17.86 ± 1.75 ^b^	14.72 ± 2.07 ^a,b^
**GST** (mM CDN·min^−1^·mg^−1^ protein)	19.61 ± 3.23 ^a^	19.32 ± 2.64 ^a^	18.86 ± 3.91 ^a^	25.20 ± 3.47 ^a^	18.58 ± 3.44 ^a^
**LDH** (mM NADH oxidated·min^−1^·mg^−1^ protein)	11.36 ± 4.78 ^a^	7.07 ± 2.38 ^a^	6.10 ± 2.56 ^a^	6.56 ± 2.82 ^a^	6.97 ± 1.060 ^a^

**Table 3:** **Cellular damage** (lipid peroxidation) **and redox cellular state** (GSH–Glutathione reduced, GSSG–Glutathione oxidised) **evaluation of liver and intestinal epithelial cells collected from mice exposed to PE MPs**. ***CTL*:** Animals fed standard chow diet without MPs; ***PE–L*:** Animals fed standard chow diet with non-fluorescent PE MPs at 0.002% (*w*/*w*); ***PE–H*:** Animals fed standard chow diet with non-fluorescent PE MPs at 0.006% (*w*/*w*); ***f-PE–L*:** Animals fed standard chow diet with fluorescent PE MPs at 0.002% (*w*/*w*); ***f-PE–H*:** Animals fed standard chow diet with fluorescent PE MPs at 0.006% (*w*/*w*). In the table, values with different letters indicate statistically significant differences. Values are presented as means ± SD (*n* = 6, per group), with two replicates.

	*CTL*	*PE–L*	*PE–H*	*f-PE–L*	*f-PE–H*
***Liver***					
**Lipid Peroxidation**					
*Cellular Fraction* (µM MDA·mg^−1^ protein)	0.450 ± 0.253 ^a^	0.427 ± 0.247 ^a,b^	0.306 ± 0.181 ^a^	0.533 ± 0.219 ^a^	1.024 ± 0.159 ^b^
*Mitochondrial Fraction* (µM MDA·mg^−1^ protein)	0.133 ± 0.034 ^a^	0.172 ± 0.058 ^a^	0.241 ± 0.047 ^a^	0.287 ± 0.062 ^a^	0.279 ± 0.189 ^a^
**Redox cellular state**					
*GSH/GSSG*	1.734 ± 0.319 ^a,b^	1.222 ± 0.356 ^a,b^	1.374 ± 0.220 ^a^	1.393 ± 0.334 ^a,b^	1.924 ± 0.233 ^b^
***Intestinal*** ***Epithelial Cells***					
**Lipid Peroxidation**					
*Cellular Fraction* (µM MDA·mg^−1^ protein)	0.501 ± 0.133 ^a^	0.559 ± 0.011 ^a^	0.546 ± 0.026 ^a^	0.567 ± 0.252 ^a^	0.2456 ± 0.058 ^a^
**Redox cellular state**					
*GSH/GSSG*	0.450 ± 0.067 ^a^	0.501 ± 0.036 ^a,b^	0.589 ± 0.093 ^b,c^	0.724 ± 0.067 ^c^	0.596 ± 0.081 ^b,c^

**Table 4:** **General characteristics of the total lipid derived from hepatic mitochondrial fractions of mice exposed to MPs**. ***CTL*:** Animals fed standard chow diet without MPs; ***PE–L*:** Animals fed standard chow diet with non-fluorescent PE MPs at 0.002% (*w*/*w*); ***PE–H*:** Animals fed standard chow diet with non-fluorescent PE MPs at 0.006% (*w*/*w*); ***f-PE–L*:** Animals fed standard chow diet with fluorescent PE MPs at 0.002% (*w*/*w*); ***f-PE–H*:** Animals fed standard chow diet with fluorescent PE MPs at 0.006% (*w*/*w*). In the table, values with different letters indicate statistically significant differences. Values are presented as means ± SD (*n* = 6, per group).

	*CTL*	*PE–L*	*PE–H*	*f-PE–L*	*f-PE–H*
Fatty acid					
**SFA** (relative abundance %)	50.58 ± 3.59 ^a^	46.46 ± 2.96 ^a^	50.62 ± 1.46 ^a^	48.96 ± 1.35 ^a^	52.17 ± 2.08 ^a^
**MUFA** (relative abundance %)	21.73 ± 2.20 ^a^	19.44 ± 3.57 ^a,b^	15.01 ± 1.42 ^b^	17.63 ± 1.22 ^a^	13.19 ± 1.22 ^b^
**PUFA** (relative abundance %)	32.54 ± 4.28 ^a^	35.79 ± 5.69 ^a^	33.55 ± 1.42 ^a^	33.41 ± 1.06 ^a^	34.25 ± 2.08 ^a^
***n*-6 PUFA** (relative abundance %)	32.30 ± 4.21 ^a^	32.94 ± 1.83 ^a^	33.44 ± 1.44 ^a^	33.30 ± 1.06 ^a^	34.15 ± 2.10 ^a^
***n*-3 PUFA** (relative abundance %)	0.17 ± 0.03 ^a^	0.11 ± 0.01 ^b^	0.11 ± 0.01 ^b^	0.11 ± 0.01 ^b^	0.08 ± 0.01 ^b^
***n*** **-3/*n*-6**	0.0072 ± 0.0018 ^a^	0.0034 ± 0.00037 ^b^	0.0032 ± 0.00045 ^b^	0.0032 ± 0.00062 ^b^	0.0026 ± 0.00045 ^b^
**PUFA/SFA**	0.61 ± 0.06 ^a^	0.70 ± 0.05 ^a^	0.66 ± 0.03 ^a^	0.68 ± 0.04 ^a^	0.66 ± 0.06 ^a^
Peroxidability index (**PI**)	0.78 ± 0.12 ^a^	0.81 ± 0.12 ^a^	0.76 ± 0.03 ^a^	0.78 ± 0.03 ^a^	0.80 ± 0.11 ^a^
Double bond index (**DBI**)	1.10 ± 0.10 ^a^	1.21 ± 0.14 ^a^	1.11 ± 0.02 ^a^	1.15 ± 0.03 ^a^	1.16 ± 0.14 ^a^

## Data Availability

The sequence datasets generated and analysed in this study have been deposited at NCBI (SRR36199943 to SRR36199954) and are available under Bioproject PRJNA1369809 (https://www.ncbi.nlm.nih.gov/sra/PRJNA1369809 (available on 31 May 2026)).

## Supplementary Materials

The following supporting information can be downloaded at: https://www.mdpi.com/article/10.3390/toxics14050386/s1. Method S1: Diet preparation; Method S2: Preservation of tissue samples for high-resolution respirometry; Method S3: Isolation of intestinal epithelial cells (IECs) suspension from the whole intestine; Figure S1: Characterisation of PE MPs. (A) SEM image of f-PE MPs at 200× magnification; (B) SEM image of f-PE MPs at 1000× magnification; (C) Size distribution of f-PE MPs; (D) SEM image of PE MPs at 200× magnification; (E) SEM image of PE MPs at 1000× magnification; (F) Size distribution of PE MPs; (G) FTIR spectrum of PE MPs; (H) FTIR spectrum of f-PE MPs; Figure S2: Impact of PE-MPs on animals’ food (A) and water (B) consumption, and ponderal weight gain (C). Animals’ weight, water consumption, and food intake were recorded weekly throughout the experiment. CTL–Animals fed standard chow diet without MPs; PE–L: Animals fed standard chow diet with non-fluorescent PE MPs at 0.002% (*w*/*w*); PE–H: Animals fed standard chow diet with non-fluorescent PE MPs at 0.006% (*w*/*w*); f-PE–L: Animals fed standard chow diet with fluorescent PE MPs at 0.002% (*w*/*w*); f-PE–H: Animals fed standard chow diet with fluorescent PE MPs at 0.006% (*w*/*w*). Values are presented as mean ± SD (*n* = 6, per group) and expressed as arbitrary units. Statistical comparisons between groups are indicated by connecting brackets in the figure. Each bracket represents a predefined pairwise comparison (* *p* < 0.05); Figure S3: Representative images of mice livers exposed to PE MPs 0.006% (A), presenting focal apoptotic hepatocytes, with hypereosinophilic cytoplasm, and f-PE MPs 0.002% (B), with focal necrosis associated with inflammation (focal group of contiguous cells with cell swelling, loss of cellular detail and neutrophils). CTL–Animals fed standard chow diet without MPs; PE–L: Animals fed standard chow diet with non-fluorescent PE MPs at 0.002% (*w*/*w*); PE–H: Animals fed standard chow diet with non-fluorescent PE MPs at 0.006% (*w*/*w*); f-PE–L: Animals fed standard chow diet with fluorescent PE MPs at 0.002% (*w*/*w*); f-PE–H: Animals fed standard chow diet with fluorescent PE MPs at 0.006% (*w*/*w*); Table S1: Main absorption bands of non-fluorescent polyethylene microplastics (PE MPs) and fluorescent polyethylene microplastics (f-PE MPs) and their assignments. Table S2: Effect of fluorescent and non-fluorescent polyethylene microplastics on mean relative organ weight. CTL–Animals fed standard chow diet without MPs; PE–L: Animals fed standard chow diet with nonfluorescent PE MPs at 0.002% (*w*/*w*); PE–H: Animals fed standard chow diet with non-fluorescent PE MPs at 0.006% (*w*/*w*); f-PE–L: Animals fed standard chow diet with fluorescent PE MPs at 0.002% (*w*/*w*); f-PE–H: Animals fed standard chow diet with fluorescent PE MPs at 0.006% (*w*/*w*). Values are presented as mean ± SD (*n* = 6, per group) and expressed as arbitrary units. Statistical comparisons between groups are indicated by connecting brackets in the figure. Each bracket represents a predefined pairwise comparison (* *p* < 0.05); Table S3: Number and percentage of animals with hepatic histologic lesions and respective score. CTL–Animals fed standard chow diet without MPs; PE–L: Animals fed standard chow diet with non-fluorescentPE MPs at 0.002% (*w*/*w*); PE–H: Animals fed standard chow diet with non-fluorescent PE MPs at 0.006% (*w*/*w*); f-PE–L: Animals fed standard chow diet with fluorescent PE MPs at 0.002% (*w*/*w*); f-PE–H: Animals fed standard chow diet with fluorescent PE MPs at 0.006% (*w*/*w*). In the table, values with different letters indicate statistically significant differences.
