# Supplementary material for: Hepatic Mitochondrial Dysfunction and Gut Dysbiosis Induced by Polyethylene Microplastics in FVB/n Mice: A Comparative Study of Fluorescent and Non-Fluorescent Particles
Source: Toxics. 2026 Apr 30;14(5):386. doi: 10.3390/toxics14050386 (PMC13211033; doi:10.3390/toxics14050386)
Supplement: Supplementary file 1 [file toxics-14-00386-s001.zip › toxics-4246159-supplementary.pdf]

Supplementary Material

Details for Material and Methods

Method S1 – *Diet preparation*

The standard chow diet was minced, and microplastics were weighed and added to the mixture. The new diet pellets (4.2 mm diameter) were prepared using an industrial mixer (CPM Europe, model C-300, Zaandam, The Netherlands) by adding 5 % (v/w) H_2O to the mixture. All the batches prepared were placed in an oven at 50 °C for 24h. The diet was then stored at 4 °C until further use.

Method S2 – *Preservation of tissue samples for high-resolution respirometry*

Livers were preserved following a similar protocol described by Garcia-Roche 2018. Tissue aliquots (approximately 10 mg) were immersed in 1 mL of ice-cold modified University of Wisconsin solution (20 mM histidine, 20 mM succinate, 3 mM glutathione, 1 µM Leupeptin, 2 mM glutamate, 2 mM malate, 2 mM ATP, 0.5 mM EGTA, 3 mM $MgCl_2 \cdot 6\ H_2O$, 60 mM MOPS, 20 mM Taurine, 10 mM KH_2PO_4, 20 mM HEPES, 110 mM Sucrose, 1 g/L BSA and 10 % DMSO (v/v)). DMSO was added immediately before the process. Samples were then placed in ice (2 – 4 °C) for 6 minutes and then stored at – 80 °C until further analysis.

Method S3 – *Isolation of intestinal epithelial cells (IECs) suspension from the whole intestine*

After the intestine was harvested, the tissue was rinsed thoroughly with distilled water to eliminate faeces and residual food particles. Ice-cold Dulbecco's Phosphate-Buffered Saline (DPBS) was added to the lumen of the intestine and stored at 4 ºC overnight. The DPBS from inside the intestine was collected, and the tissues were rinsed with fresh DPBS. Next, the lumen was gently scraped using forceps, simultaneously squeezing the intestine to displace epithelial cells. The cell suspension containing epithelial cells and luminal material was washed twice by centrifuging at 1.000 *g*, 5 min., 4 ºC. The pelleted cells were resuspended in DPBS and stored at – 80 ºC for downstream analysis.

Results

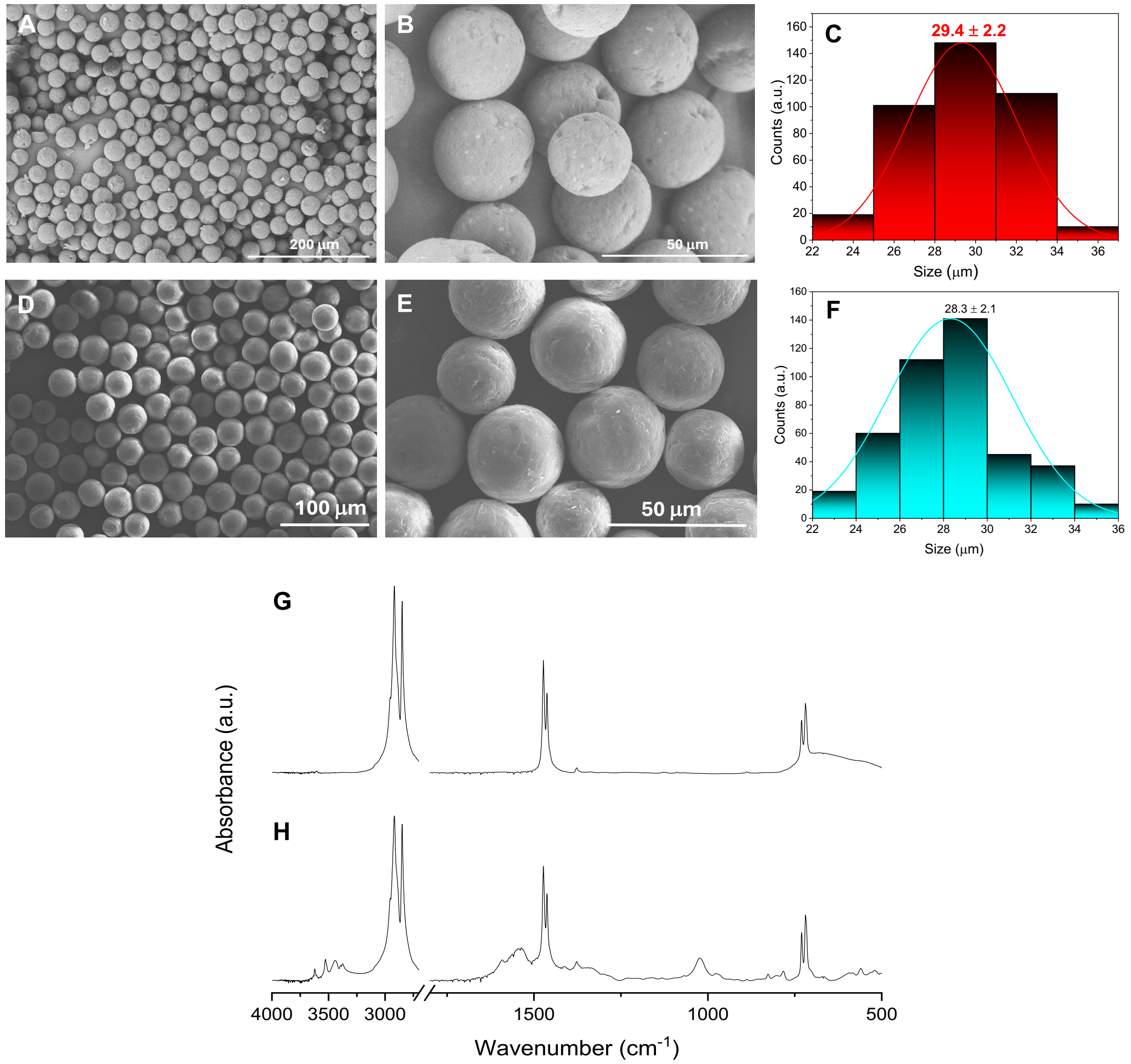

Figure S1 – **Characterisation of PE MPs. (A)** SEM image of f-PE MPs at 200 × magnification; **(B)** SEM image of f-PE MPs at 1000 × magnification; **(C)** Size distribution of f-PE MPs; **(D)** SEM image of PE MPs at 200 × magnification; **(E)** SEM image of PE MPs at 1000 × magnification; **(F)** Size distribution of PE MPs; **(G)** FTIR spectrum of PE MPs; (**H**) FTIR spectrum of f-PE MPs;

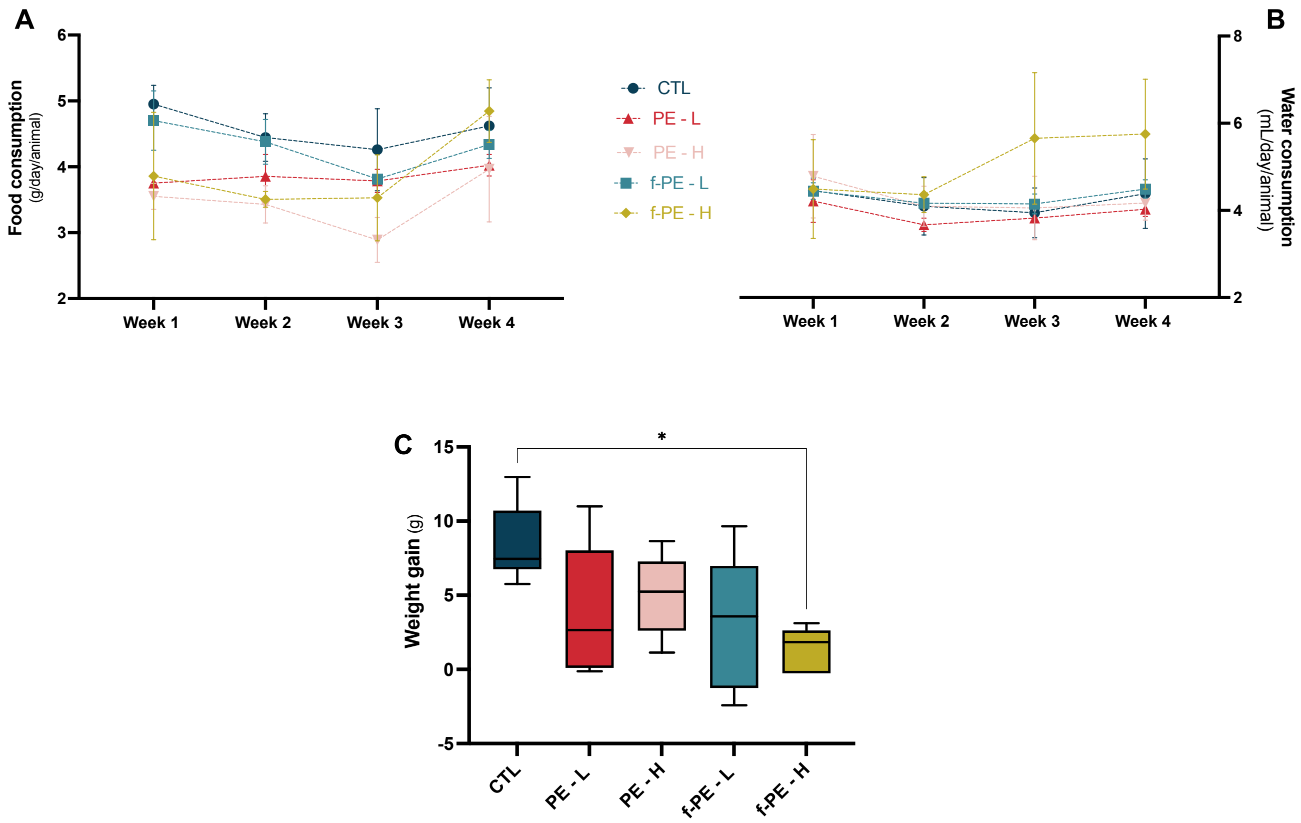

Figure S2 – **Impact of PE-MPs on animals' food (A) and water (B) consumption, and ponderal weight gain** (**C**). Animals' weight, water consumption, and food intake were recorded weekly throughout the experiment. ***CTL*** – Animals fed standard chow diet without MPs; ***PE – L:*** Animals fed standard chow diet with non-fluorescent PE MPs at 0.002 % (w/w); ***PE – H:*** Animals fed standard chow diet with non-fluorescent PE MPs at 0.006 % (w/w); ***f-PE – L:*** Animals fed standard chow diet with fluorescent PE MPs at 0.002 % (w/w); ***f-PE – H:*** Animals fed standard chow diet with fluorescent PE MPs at 0.006 % (w/w). Values are presented as mean ± SD (n=6, per group) and expressed as arbitrary units. Statistical comparisons between groups are indicated by connecting brackets in the figure. Each bracket represents a predefined pairwise comparison (*p < 0.05)

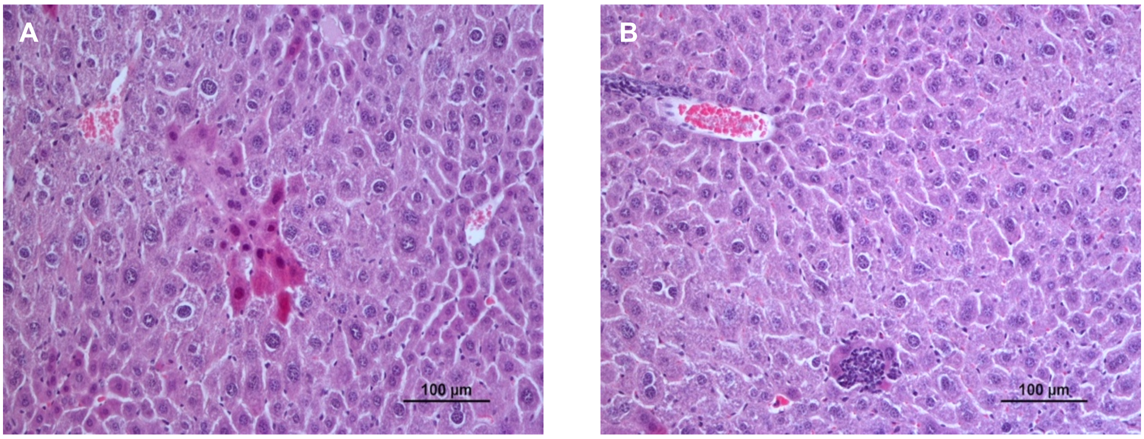

Figure S3 – Representative images of mice livers exposed to PE MPs 0.006 % **(A)**, presenting focal apoptotic hepatocytes, with hypereosinophilic cytoplasm, and f-PE MPs 0.002 % **(B)**, with focal necrosis associated with inflammation (focal group of contiguous cells with cell swelling, loss of cellular detail and neutrophils). ***CTL*** – Animals fed standard chow diet without MPs; ***PE – L:*** Animals fed standard chow diet with non-fluorescent PE MPs at 0.002 % (w/w); ***PE – H:*** Animals fed standard chow diet with non-fluorescent PE MPs at 0.006 % (w/w); ***f-PE – L:*** Animals fed standard chow diet with fluorescent PE MPs at 0.002 % (w/w); ***f-PE – H:*** Animals fed standard chow diet with fluorescent PE MPs at 0.006 % (w/w).

Samples	Experimental band (cm^{-1})	Attribution
f-PE MPs; PE MPs	2921	$-CH_2$ asymetric stretching
f-PE MPs; PE MPs	2847	$-CH_2$ asymetric stretching
f-PE MPs; PE MPs	1472	$-CH_2$ bending deformation
f-PE MPs; PE MPs	1463	$-CH_2$ bending deformation
f-PE MPs; PE MPs	1377	$-CH_3$ symetric deformation
f-PE MPs	1023	-
f-PE MPs	978	-
f-PE MPs; PE MPs	730	Rocking deformation
f-PE MPs; PE MPs	717	Rocking deformation
f-PE MPs; PE MPs	559	-

Table S1. Main absorption bands of non-fluorescent polyethylene microplastics (PE MPs) and fluorescent polyethylene microplastics (f-PE MPs) and their assignments.

	GROUP	RELATIVE ORGANS WEIGHT (%)
**LIVER**	CTL	4.220 ± 0.558[a]
	PE – L	4.589 ± 0.272[a]
	PE – H	4.122 ± 0.256[a]
	f-PE – L	4.350 ± 0.352[a]
	f-PE – H	4.796 ± 0.494[a]
**KIDNEY**	CTL	0.641 ± 0.0297[a]
	PE – L	0.698 ± 0.0477[a]
	PE – H	0.697 ± 0.0443[a]
	f-PE – L	0.683 ± 0.0301[a]
	f-PE – H	0.792 ± 0.0869[b]
**TESTICLE**	CTL	0.250 ± 0.0170[a]
	PE – L	0.262 ± 0.0250[a]
	PE – H	0.307 ± 0.1567[b]
	f-PE – L	0.280 ± 0.0413[a]
	f-PE – H	0.330 ± 0.0195[b]
**HEART**	CTL	0.502 ± 0.0505[a,b]
	PE – L	0.50 ± 0.0317[a]
	PE – H	0.430 ± 0.0472[a]
	f-PE – L	0.452 ± 0.0732 [a]
	f-PE – H	0.608 ± 0.1261[b]

Table S2 – Effect of fluorescent and non-fluorescent polyethylene microplastics on mean relative organ weight. ***CTL*** – Animals fed standard chow diet without MPs; ***PE – L:*** Animals fed standard chow diet with non-fluorescent PE MPs at 0.002 % (w/w); ***PE – H:*** Animals fed standard chow diet with non-fluorescent PE MPs at 0.006 % (w/w); ***f-PE – L:*** Animals fed standard chow diet with fluorescent PE MPs at 0.002 % (w/w); ***f-PE – H:*** Animals fed standard chow diet with fluorescent PE MPs at 0.006 % (w/w). Values are presented as mean ± SD (n=6, per group) and expressed as arbitrary units. Statistical comparisons between groups are indicated by connecting brackets in the figure. Each bracket represents a predefined pairwise comparison (*$p < 0.05$)

HAI	CTL	PE – L	PE – H	f-PE – L	f-PE – H
**Piecemal necrosis**					
Score 0	10/10 (100%)[a]	9/9 (100%)[a]	9/9 (100%)[a]	10/10 (100%)[a]	8/9 (88.9%)[a]
Score 1	0/10 (0%)[a]	0/9 (0%)[a]	0/9 (0%)[a]	0/10 (0%)[a]	1/9 (11.11%)[a]
Score 2	0/10 (0%)[a]	0/9 (0%)[a]	0/9 (0%)[a]	0/10 (0%)[a]	0/9 (0%)[a]
Score 3	0/10 (0%)[a]	0/9 (0%)[a]	0/9 (0%)[a]	0/10 (0%)[a]	0/9 (0%)[a]
Score 4	0/10 (0%)[a]	0/9 (0%)[a]	0/9 (0%)[a]	0/10 (0%)[a]	0/9 (0%)[a]
**Confluent necrosis**					
Score 0	10/10 (100%)[a]	9/9 (100%)[a]	9/9 (100%)[a]	10/10 (100%)[a]	9/9 (100%)[a]
Score 1	0/10 (0%)[a]	0/9 (0%)[a]	0/9 (0%)[a]	0/10 (0%)[a]	0/9 (0%)[a]
Score 2	0/10 (0%)[a]	0/9 (0%)[a]	0/9 (0%)[a]	0/10 (0%)[a]	0/9 (0%)[a]
Score 3	0/10 (0%)[a]	0/9 (0%)[a]	0/9 (0%)[a]	0/10 (0%)[a]	0/9 (0%)[a]
Score 4	0/10 (0%)[a]	0/9 (0%)[a]	0/9 (0%)[a]	0/10 (0%)[a]	0/9 (0%)[a]
Score 5	0/10 (0%)[a]	0/9 (0%)[a]	0/9 (0%)[a]	0/10 (0%)[a]	0/9 (0%)[a]
Score 6	0/10 (0%)[a]	0/9 (0%)[a]	0/9 (0%)[a]	0/10 (0%)[a]	0/9 (0%)[a]
**Focal necrosis, apoptosis, focal inflammation**					
Score 0	7/10 (70%)[a]	3/9 (33.3%)[a,b]	1/9 (11.1%)[b]	1/10 (10%)[b]	3/9 (33.3%)[a,b]
Score 1	3/10 (30%)[a]	6/9 (66.7%)[a]	5/9 (55.6%)[a]	7/10 (70%)[a]	3/9 (33.3%)[a]
Score 2	0/10 (0%)[a]	0/9 (0%)[a,b]	2/9 (22.2%)[a,b]	2/10 (20%)[a,b]	3/9 (33.3%)[b]
Score 3	0/10 (0%)[a]	0/9 (0%)[a]	1/9 (11.1%)[a]	0/10 (0%)[a]	0/9 (0%)[a]
Score 4	0/10 (0%)[a]	0/9 (0%)[a]	0/9 (0%)[a]	0/10 (0%)[a]	0/9 (0%)[a]
**Portal Inflammation**					
Score 0	5/10 (50%)[a]	4/9 (44.4%)[a]	5/9 (55.6%)[a]	4/10 (40%)[a]	2/9 (22.2%)[a]
Score 1	2/10 (20%)[a]	5/9 (55.6%)[a,b]	3/9 (33.3%)[a,b]	4/10 (40%)[a,b]	6/9 (66.7%)[b]
Score 2	3/10 (30%)[a]	0/9 (0%)[a]	1/9 (11.1%)[a]	2/10 (20%)[a]	1/9 (11.1%)[a]
Score 3	0/10 (0%)[a]	0/9 (0%)[a]	0/9 (0%)[a]	0/10 (0%)[a]	0/9 (0%)[a]
Score 4	0/10 (0%)[a]	0/9 (0%)[a]	0/9 (0%)[a]	0/10 (0%)[a]	0/9 (0%)[a]
**Necroinflammatory score**					
Score 0	4/10 (40%)[a]	2/9 (22.2%)[a,b]	1/9 (11.1%)[a,b]	0/10 (0%)[b]	0/9 (0%)[b]
Score 1	2/10 (20%)[a]	3/9 (33.3%)[a]	2/9 (22.2%)[a]	4/10 (40%)[a]	3/9 (33.3%)[a]
Score 2	3/10 (30%)[a]	4/9 (44.4%)[a]	4/9 (44.4%)[a]	3/10 (30%)[a]	3/9 (33.3%)[a]
Score 3	1/10 (10%)[a]	1/9 (11.1%)[a]	1/9 (11.1%)[a]	3/10 (30%)[a]	3/9 (33.3%)[a]
Score 4	0/10(0%)[a]	1/9 (11.1%)[a]	1/9 (11.1%)[a]	0/10(0%)[a]	0/9 (0%)[a]
Score 5	0/10 (0%)[a]	0/9 (0%)[a]	0/9 (0%)[a]	0/10 (0%)[a]	0/9 (0%)[a]
Score 6-18	0/10 (0%)[a]	0/9 (0%)[a]	0/9 (0%)[a]	0/10 (0%)[a]	0/9 (0%)[a]

Table S3 – **Number and percentage of animals with hepatic histologic lesions and respective score.** ***CTL*** – Animals fed standard chow diet without MPs; ***PE – L:*** Animals fed standard chow diet with non-fluorescent PE MPs at 0.002 % (w/w); ***PE – H:*** Animals fed standard chow diet with non-fluorescent PE MPs at 0.006 %

(w/w); ***f-PE – L:*** Animals fed standard chow diet with fluorescent PE MPs at 0.002 % (w/w); ***f-PE – H:*** Animals fed standard chow diet with fluorescent PE MPs at 0.006 % (w/w). In the table, values with different letters indicate statistically significant differences.
